# Bacterial fitness landscapes stratify based on proteome allocation associated with discrete aero-types

**DOI:** 10.1371/journal.pcbi.1008596

**Published:** 2021-01-19

**Authors:** Ke Chen, Amitesh Anand, Connor Olson, Troy E. Sandberg, Ye Gao, Nathan Mih, Bernhard O. Palsson

**Affiliations:** 1 Department of Bioengineering, University of California, San Diego, La Jolla, California, United States of America; 2 Division of Biological Sciences, University of California, San Diego, La Jolla, California, United States of America; 3 Novo Nordisk Foundation Center for Biosustainability, Technical University of Denmark, Lyngby, Denmark; The Pennsylvania State University, UNITED STATES

## Abstract

The fitness landscape is a concept commonly used to describe evolution towards optimal phenotypes. It can be reduced to mechanistic detail using genome-scale models (GEMs) from systems biology. We use recently developed GEMs of Metabolism and protein Expression (ME-models) to study the distribution of *Escherichia coli* phenotypes on the rate-yield plane. We found that the measured phenotypes distribute non-uniformly to form a highly stratified fitness landscape. Systems analysis of the ME-model simulations suggest that this stratification results from discrete ATP generation strategies. Accordingly, we define “aero-types”, a phenotypic trait that characterizes how a balanced proteome can achieve a given growth rate by modulating 1) the relative utilization of oxidative phosphorylation, glycolysis, and fermentation pathways; and 2) the differential employment of electron-transport-chain enzymes. This global, quantitative, and mechanistic systems biology interpretation of fitness landscape formed upon proteome allocation offers a fundamental understanding of bacterial physiology and evolution dynamics.

## Author summary

Genome-scale models enable quantitative prediction of bacterial phenotypes and a fine-grained description of the underlying optimal proteome allocation. Thus, we can now analyze the phenotypic potential of a large number of *Escherichia coli* genotypes grown under different conditions, which leads to the discovery of a stratified distribution of phenotypes. The observed distribution is determined by distinct ATP generation strategies, defined as “aero-types”, associated with optimal proteome allocation modulated upon differential usage of the electron-transport-chain enzymes. This mechanistic approach offers us a genome-scale understanding of the fitness landscape, and a fundamental interpretation of bacterial physiology and evolution dynamics.

## Introduction

Sewall Wright’s fitness landscape [1] represented an early attempt to illustrate the complex genotype-fitness relationship in a graphical manner that allows an easy conceptualization of evolutionary dynamics. Technology developments in diverse fields including mutagenesis, microbial evolution experiments, and high-throughput DNA sequencing methods have now turned this concept from a metaphor into real data that allows for the reconstruction of the empirical fitness landscapes [2–4]. Two classes of such landscapes are usually studied to examine how natural selection may drive a population to the top of a fitness peak. The first one is constructed based on discreteness of the protein sequences, where evolution is modeled as movement through the evolutionary intermediates along feasible mutational pathways. This discrete representation is useful for estimating the probability of evolutionary outcomes [5] and demonstrating how molecular and epistatic interactions limit the number of accessible evolutionary paths [6–9]. However, experimental exploration and subsequent mathematical modeling of the fitness landscape is limited to a well-characterized posterior selection of mutations in an intrinsically high-dimensional genotype space. The second model specifies the phenotype-fitness relationship in a continuous and multivariate phenotypic space. It is capable of fitting variation in landscape structure across many species and environments [10–12], but with an impaired ability to relate fitness change directly to a specific genetic and molecular mechanism.

These well-studied fitness landscape models, whether discrete or continuous, address evolutionary dynamics towards an optimal phenotype based on the rare beneficial mutations that arise historically or in the course of microbial evolution experiments [13]. Directed evolution expedites the search for beneficial mutations in the high-dimensional sequence space by enforcing selection in the desired function and discarding those variants with no improvement. This powerful technique is capable of elucidating the molecular mechanisms of adaptation and evolutionary tradeoff in protein properties [14] under diverse environments [15], therefore greatly enriching our understanding of the adaptive trajectory. However, fitness effects for the majority of mutations that arise in nature are neutral, slightly deleterious, and slightly beneficial [16]. The distribution of the fitness effects of these spontaneous mutations in natural bacterial populations remains unclear.

An alternative approach to explore the fitness landscape and phenotypic distribution comes from the solution space of a genome-scale metabolic model (M-model) [17, 18]. Genome-scale models explicitly compute how the system-level optimization of organismal fitness is achieved through natural evolution while considering the constraints on as many factors as possible. These include the metabolic burden, resource allocation, and the interactions between gene and cellular environment [19]. The models’ ability to predict phenotypes and rapidly screen millions of genotypes allows for the exploration of the change in an optimal solution space upon gene deletion, providing valuable insight into the impact of gene essentiality [20] under diverse conditions [21], plasticity and robustness of metabolic networks [22], and the effect of epistasis interactions on the fitness distribution [23, 24].

Expansion of the M-models to include constraints on the cost of protein biosynthesis has been improving the accuracy of phenotypic predictions for different organisms under various environments [25–28]. The genome-scale models of metabolism and protein expression (ME-models) for *E. coli*, in particular, explicitly incorporate the full reconstruction of transcription and translation pathways to allow for quantitative predictions of proteome allocation at the gene level [29–31] and the ability to predict evolutionary outcomes [32]. A more recent development further takes into account the temperature-dependent catalytic efficiency and thermostability of all enzymes in the ME-model (FoldME-model [33]), enabling an explicit formulation of the effect of a gene mutation in contrast to a direct gene deletion. This final improvement provides us with the opportunity to evaluate the phenotypic distribution of natural *E. coli* populations on a fitness landscape.

Here, we assemble and analyze large amounts of *E. coli* phenotypic growth data in the rate-yield plane and find consistent non-uniformity in the fitness distribution. Both computationally and experimentally determined phenotypes display multiple distinct phenotypic categories that distribute in stripes on the rate-yield plane and form a landscape with a “stratified” topology. We then show, by detailed analysis of metabolic fluxes and protein expression, that the stratified topography of this phenotypic fitness landscape can be fully described by the energy production strategy, which in turn is determined by a balance between proteome allocation cost and the metabolic efficiency of ATP production. Interestingly, we find that a simple quantity—the fraction of total ATP that is generated by the ATP synthase (*f*_*ATPS*_)—is capable of outlining the stratification. Consequently, we define *E. coli* “aero-types” based on the multimodal distribution of *f*_*ATPS*_ modulated through the discrete usage of electron-transport-chain enzymes. An aero-type not only describes the cellular respiratory behavior, but also indicates the associated metabolic state and proteomic compositions. Finally, we discuss how the aero-type, as an effective fitness descriptor, can be used to address important biological questions such as the predictability of microbial evolution and the interpretation of the rate-yield tradeoff.

## Results

### A stratified structure in the *E. coli* phenotypic fitness landscape defined on the rate-yield plane

We used the most fundamental bacterial growth parameters, the biomass yield (*Y*) and substrate uptake rate (*q*), to span the phenotypic space for *E. coli* (Materials and methods). To gain a comprehensive view of the fitness distribution, we first compiled a compendium of experimental growth phenotypes from literature augmented with measurements obtained from our adaptive laboratory evolution (ALE) experiments (Materials and methods and references therein). This data set (*n* = 199) includes characterizations of different naturally occurring *E. coli* strains, evolved gene knock-out mutants, and growth under various nutrient conditions. It is immediately noticeable that both the high and low yield regions are densely populated, yet the regions in between (0.2 < *Y* < 0.3*gDW*/*g*) are almost empty (S1 Fig).

Is the observed non-uniform distribution of the rate-yield phenotype a result of insufficient sampling from experimental data, or a fundamental property determined by the design of a cell’s genome and metabolic network? To answer this question, we used the FoldME model [33] to compute the phenotypic fitness for a large number of in silico strains that sample the genetic variations of the naturally occurring *E. coli* genomes (Materials and methods). To implement such strain sampling, we first selected genes for mutation according to the calculated frequency of fixed mutations for each gene (S2 Fig). Then, we determined the molecular effect of the selected mutation by varying the selected enzyme’s catalytic efficiency (*k*_*eff*_) and thermal stability (Δ*G*) by a random but small amount (see Materials and Methods for more details). Finally, growth of the sampled strains was simulated under glucose minimal media with temperature perturbations from 25°C to 46°C to take into account the effect of both genetic mutations and environmental changes.

**Fig 1:**
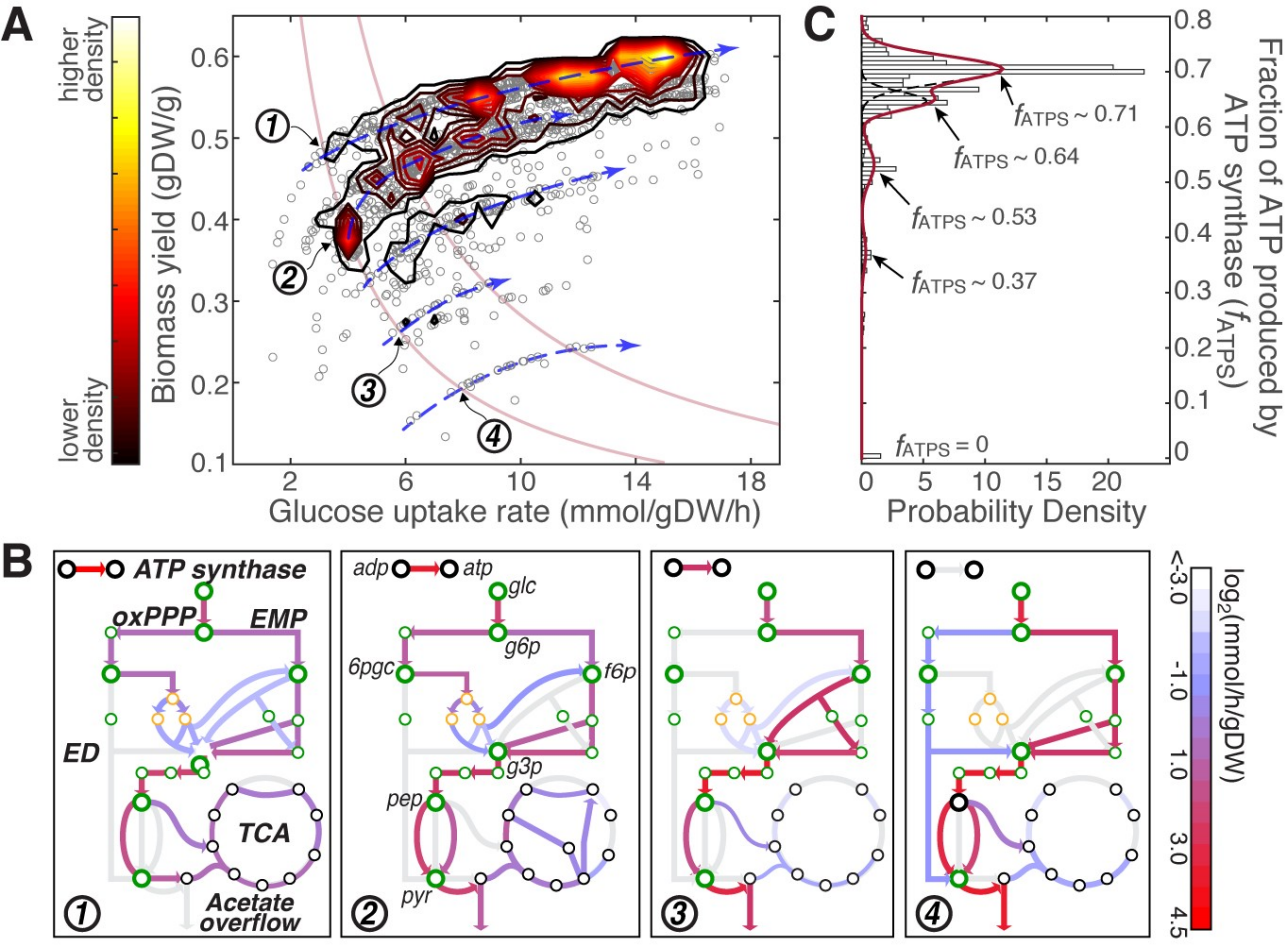
Multimodal distribution of *f*_*ATPS*_ determines the discrete metabolic state and the stratification of phenotypic landscape. (A) Fitness effect calculated from 2,200 in silico *E. coli* strains shows a stratified phenotype distribution on the rate-yield plane. Blue arrows indicate the populated regions, within which metabolic flux distribution remains relatively constant. Two example *μ*-isoclines are highlighted by red solid lines. Numbers in the open circles indicate the locations of four in silico strains selected for metabolic flux analysis shown in panel B. (B) Four representative metabolic states are depicted by the flux distribution of major pathways in the central metabolism, including the glycolysis pathway (metabolites colored in green), oxidative pentose phosphate pathway (oxPPP, yellow), and the TCA cycle (black). Key metabolites indicated on the figure are, glc: glucose; g6p: D-glucose 6-phosphate; g3p: glyceraldehyde 3-phosphate; f6p: D-fructose 6-phosphate; pyr: pyruvate; 6pgc: 6-phospho-D-gluconate; pep: phosphoenolpyruvate. Calculated fluxes of each state are colored on a log scale. (C) Distribution of the computed *f*_*ATPS*_ fitted to a mixture of four Gaussian distributions. The result shows four peaks centered on 0.37, 0.53, 0.64, and 0.71. An additional peak is seen at *f*_*ATPS*_ = 0. Peaks in the multimodal distribution of *f*_*ATPS*_ are highly correlated with the populated regions on the rate-yield plane shown by the blue arrows in panel A.

The calculated fitness effects for the in silico strains were projected onto the rate-yield plane. The contour plot of a total of 2,200 sampled *E. coli* strains (Fig 1A) nicely confirms the non-uniform distribution observed from the experimental data. More importantly, it offers a characteristic representation for the “phenotypic fitness landscape”, in which growth phenotypes densely cluster along a few hyperbolic lines on the rate-yield plane (indicated by the blue arrows) but rarely fall in between these stratified density peaks.

### The metabolic location of ATP production stratifies the phenotypic fitness landscape

To explain the observed stratification in phenotype distribution, we first examined the metabolic features characterizing the simulated samples within each populated region on the rate-yield plane. Interestingly, solutions along the densely populated hyperbolic lines (blue arrows in Fig 1A), where *q* and *Y* are positively correlated, share similar features in their flux distributions in central metabolism (S3 Fig). On the contrary, samples along the constant growth rate lines (*μ*-isoclines, red solid lines in Fig 1A) show consistent variation in the metabolic states that correlate with shifts in the rate-yield phenotype.

Specifically, as *Y* decreases along a *μ*-isocline, the following changes in the metabolic state can be identified through principal component analysis (Fig 1B and S3 Fig): 1) the amount of ATP produced by ATP synthase decreases; 2) flux through the tricarboxylic acid (TCA) cycle decreases; 3) total flux through the glycolysis pathway increases; 4) acetate secretion increases; and 5) the overall metabolic complexity, measured by the number of active reactions, decreases. We analyzed the expression data from 17 *E. coli* strains evolved under glucose minimal medium at 37°C [34] and 42°C [35], and confirmed the first two calculated trends with the positive correlation between *Y* and the total mass fraction of genes involved in TCA cycle and oxidative phosphorylation (S4 Fig).

We noticed that flux change of the energy production reactions correlated well with the shift in metabolic state and phenotypic location. Hence, we computed the fraction of total ATP produced by eight ATP-producing reactions: 1) ATP production by the ATP synthase (ATPS4rpp), and reactions catalyzed by the polyphosphate kinase (PPKr and PPK2r) in oxidative phosphorylation; 2) reactions catalyzed by the phosphoglycerate kinase (PGK) and the pyruvate kinase (PYK) in the lower glycolysis pathway; 3) the reaction catalyzed by the acetate kinase (ACKr) in mixed acid fermentation; 4) the reaction catalyzed by the succinyl-CoA synthetase (SUCOAS) in the TCA cycle; and 5) the reaction catalyzed by the ribose-phosphate diphosphokinase (PRPPS) in nucleotide biosynthesis. These quantities (*f*_*ATPS*_, *f*_*PGK*_, *f*_*ACKr*_, etc.) formed an eight-element vector that we used as the explanatory variables in a stepwise linear regression analysis. The results showed that six of the ATP production fractions could explain 89.5% of the variation in phenotypic distance (Materials and methods, S5 Fig), confirming the predictable mapping relationships between the metabolic state of ATP production and the phenotype.

Among these ATP production fractions, *f*_*ATPS*_ appears to be of particular importance. The fact that *f*_*ATPS*_ is positively correlated with *Y* and negatively correlated with *q*_*glc*_ at each specific growth rate (S6 Fig), identifies it as the metabolic origin for the observed relationship between a metabolic state and the rate-yield phenotype. The observed correlation is rooted fundamentally in the cell’s energetic and metabolic network, rather than being just a simple function of the expression of the ATP synthase (S7 Fig). Interestingly, *f*_*ATPS*_ displays a multimodal distribution that is highly correlated with the distribution of the rate-yield phenotypes. Solutions with higher *f*_*ATPS*_ values (e.g., with averages 0.71 or 0.64) are located within the top two hyperbolic bands on the rate-yield plane (Fig 1C). For these high-yield phenotypes, high-resolution ^13^C-metabolic flux data is available to estimate their *f*_*ATPS*_ values experimentally. We calculated *f*_*ATPS*_ to be ∼0.65 for *E. coli* MG1655 evolved under glucose minimal medium and ∼0.706 for *E. coli* BL21 [36], both within 1.5% difference of the peak values predicted by our simulations.

To further confirm the critical role of *f*_*ATPS*_, we tested whether the discreteness of *f*_*ATPS*_ directly gave rise to the stratified structure of the phenotypic fitness landscape. We performed strain sampling simulations where *f*_*ATPS*_ was constrained at the five predicted peak values: 0, 0.37, 0.53, 0.64, and 0.71 (Materials and methods). The results showed clearly that optimal solutions obtained at a particular *f*_*ATPS*_ were constrained within a thin hyperbolic band, where *q* and *Y* were positively correlated (S8(A) Fig). Under the same substrate supply, the higher the *f*_*ATPS*_, the higher biomass yield can be achieved, consistent with correlations shown in S6(B) Fig. This reconstructed fitness landscape fully reproduced the observed stratified phenotypic distribution.

In summary, we introduced the fraction of total ATP produced by the ATP synthase (*f*_*ATPS*_) as a simple, yet effective, quantification for the cell’s metabolic state, and key determining factor for the stratified phenotypic distribution on the rate-yield plane.

### Multimodal distribution of *f*_*ATPS*_ is constrained by proteome complexity of the ATP production pathways

The quantitative relationship between *f*_*ATPS*_ and a cell’s metabolic and phenotypic state inspired the investigation for the underlying constraints imposed on the ATP production reactions. To deduce the source of this constraint, we look for systematic differences in protein expression profiles between solutions with different *f*_*ATPS*_ values. First, we generated sampling simulations constrained to six defined growth rates at 30°C to limit uncontrolled biases from temperature-induced differences in growth rate (Materials and methods, S9(A) Fig). The result confirmed the observed relationships by reproducing the multimodal distribution centered at the same *f*_*ATPS*_ values (S9(B) Fig).

**Fig 2:**
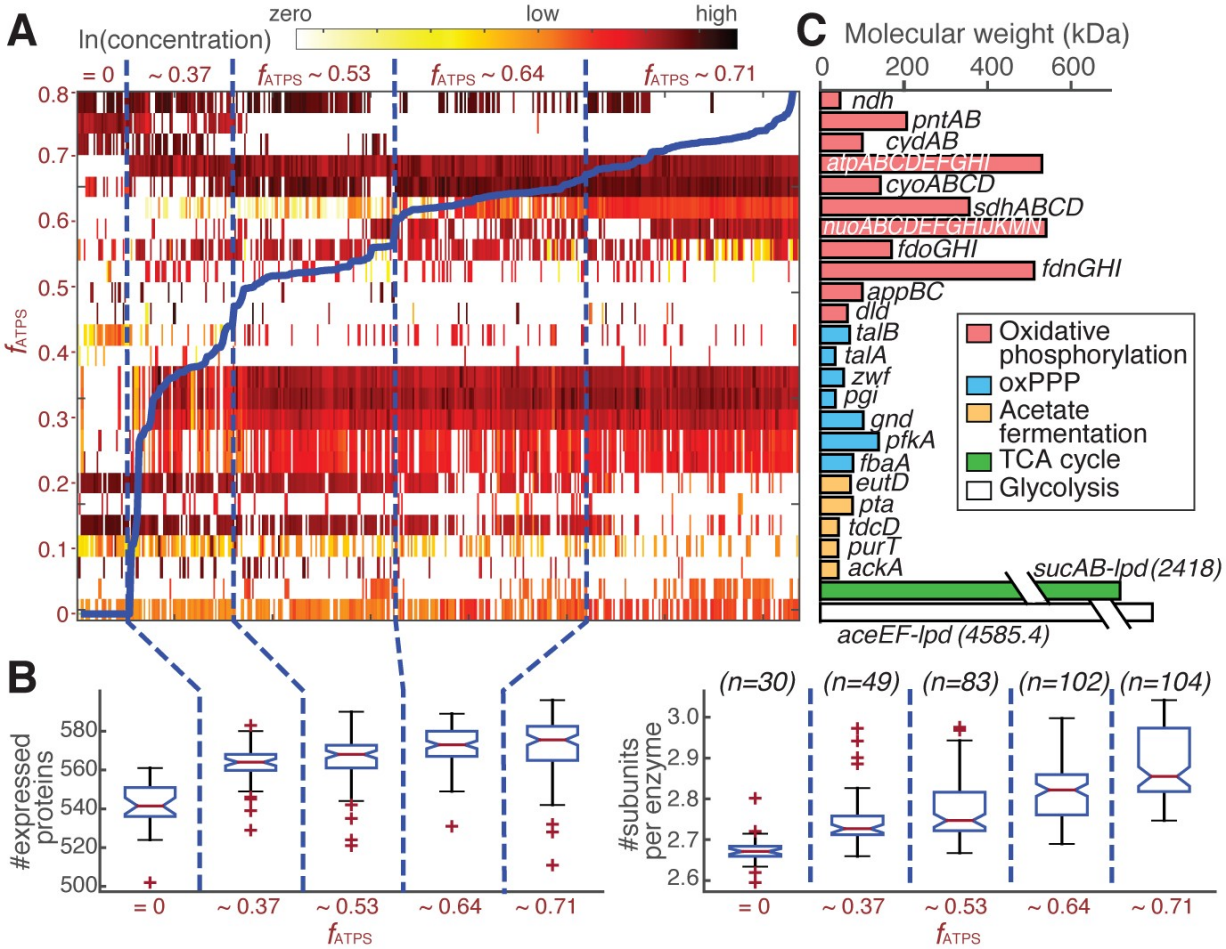
Multimodal distribution of *f*_*ATPS*_ is determined by proteome complexity. (A) Simulated concentration of the enzymes shown in panel C across 368 sampling simulations, ordered by their computed *f*_*ATPS*_ values (thick blue solid line). (B) Proteome complexity, measured by the calculated number of genes expressed (left) and the average number of subunits per enzyme (right) in the optimal solution. Both numbers increase as the calculated *f*_*ATPS*_ increases. The data is represented as box plots with the central red line showing the median, the bottom and top edges indicating the 25^*th*^ and 75^*th*^ percentiles, respedtively, and whiskers extending to 1.5 times the interquartile range. The non-overlapping notches on the boxplot show that the medians between groups differ with a 95% confidence. Number of samples in each box is indicated in parenthesis. (C) Molecular weight of the selected protein and protein complexes catalyzing reactions in the ATP-producing pathways.

Next, we order the expression profiles of the simulated strains by their computed *f*_*ATPS*_ values (Fig 2A). We find that an increase in *f*_*ATPS*_ is accompanied by a shift to a more complex proteome. The increase in proteome complexity is manifested in two ways. First, the number of genes expressed increases (Fig 2B left). For example, the pentose phosphate pathway and the multi-gene protein complexes in oxidative phosphorylation are only extensively used when aerobic respiration is turned on (*f*_*ATPS*_ > 0). Second, the average number of subunits per enzyme increases (Fig 2B right). In other words, as ATP synthase becomes responsible for a larger fraction of ATP production, the cell tends to use larger multi-domain protein complexes instead of single-gene enzymes with low molecular mass.

The switch between single-gene and multi-domain enzymes is the most obvious in oxidative phosphorylation pathways, and particularly electron transport chain (ETC) reactions (Fig 2C). For example, reduction of the quinone pool is mainly performed by the NADH dehydrogenase II Ndh at low *f*_*ATPS*_, but switches to larger protein complexes, such as the formate dehydrogenase and the NADH:quinone oxidoreductase, as *f*_*ATPS*_ increases. In the subsequent oxidation of quinol and transport of protons across the inner membrane, the smaller oxidase complex CydABX is used at low *f*_*ATPS*_, and the larger alternative CyoABCD takes over at higher *f*_*ATPS*_. We note that approximately 60% of the reactions in oxidative phosphorylation rely on one or multiple protein complexes for catalysis (S1 Table). Compared to other metabolic pathways, this high level of protein complexity is likely an evolutionary result to provide more flexibility and fine-tuning for the discrete selection of energy production strategies.

These results reveal an intricate balance between proteome complexity and the energy requirements for cell growth. As the energy demand increases, more and more enzyme complexes are necessary to achieve higher ATP yield. However, larger complexes also require significantly more metabolic resources for their biosynthesis. Thus, once activated, these enzyme complexes should be used as much as possible, inducing necessary rewiring of the metabolic network for optimal balance in proteome allocation, and shifting the ATP production strategy to the next discrete state.

### Introduction of the “aero-type” as a phenotypic trait defined based on *f*_*ATPS*_

We have shown that aerobic respiration through ATP synthase determines the cell’s metabolic state and its phenotypic location on the rate-yield plane. Accordingly, we define “aero-types” *i* to *v* to describe the five populated phenotypes represented by the five peak values of *f*_*ATPS*_ (from *f*_*ATPS*_ = 0 to *f*_*ATPS*_ ∼ 0.71) observed in the strain sampling simulations. Computationally, we compare aero-type with the P/O ratio, a commonly used parameter that describes the cellular respiratory behavior. We show that the P/O ratio outlines only the local stoichiometry of the oxidative phosphorylation pathways. Aero-type offers a more global description of cellular fitness by representing the metabolic and phenotypic state, and the proteome complexity associated with a specific energy production scheme (S1 Text and S10 Fig). Nevertheless, experimental evidence is necessary to establish the computationally defined aero-type as a practical proxy measure for the bacterial fitness.

We resorted to the characterization of genetic mutations that may trigger a switch in the aero-type. According to the comprehensive decomposition of the ETC enzyme usage shown in Fig 2 and S10 Fig, we selected two genes from the dehydrogenase (*nuoB* from the NADH dehydrogenase I and the NADH dehydronase II gene *ndh*) and two from the cytochrome oxidase (*cyoB* from the cytochrome *bo* oxidase and *cydB* from the cytochrome *bd*-I oxidase) for genetic manipulation (Materials and methods). We would expect that the removal of *ndh* would most likely switch the cell to aero-type *iv* or *v*, which have the highest *Y* and the lowest *q*_*glc*_ on the rate-yield plane. Removing *cyoB* (regardless of which NADH dehydrogenase is present) would most likely leave the cell in aero-type *i* and *ii*, with lower *Y* and higher *q*_*glc*_. The mutants depleted of *cydB* and/or *nuoB* are, in principle, still accessible to all aero-types. However, it is less likely for the Δ*nuoB* mutant to have higher *Y* and lower *q*_*glc*_, because the NADH dehydrogenase I is almost always activated for aero-type *iv* and *v*.

**Fig 3:**
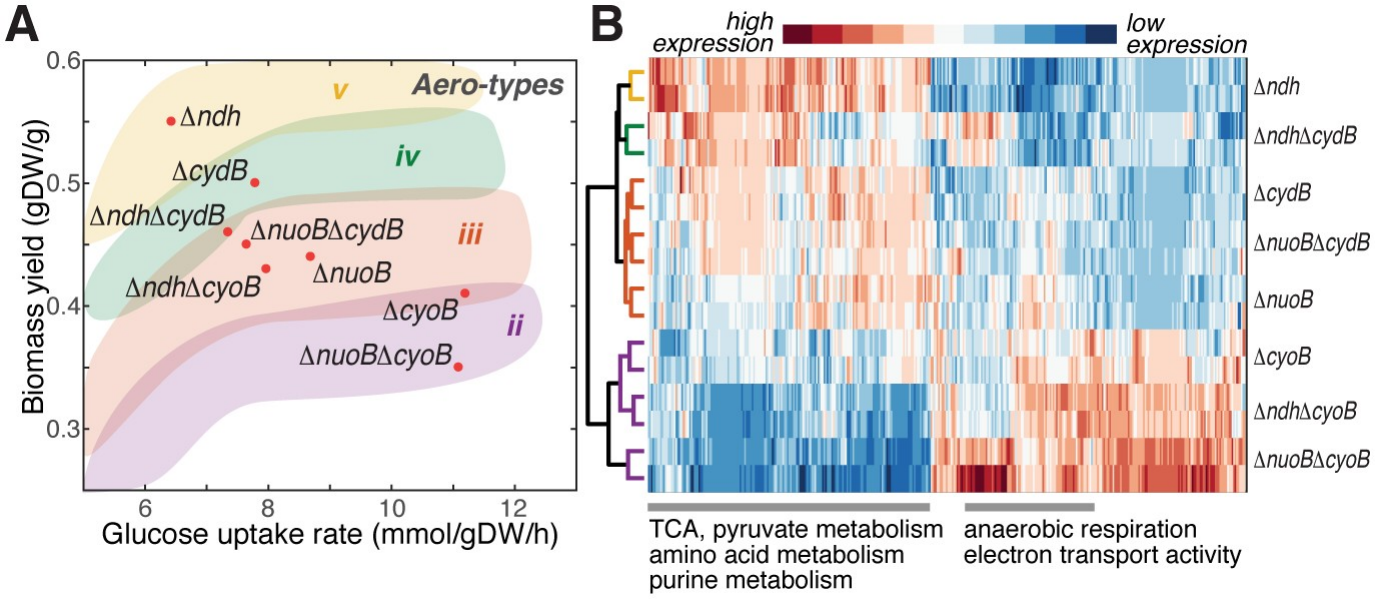
Experimental characterization of aero-type for the *E. coli* ETC-enzyme knockout strains. (A) Phenotypic characterization for the single and double ETC knockout strains on the rate-yield plane. Aero-types are assigned according to the computationally defined color-shaded area on the rate-yield plane. (B) Expression profiles for the mutant strains are shown for central metabolic genes involved in glycolysis, pyruvate pathway, pentose phosphate pathway, oxidative phosphorylation, TCA cycle, amino acid metabolism, and nucleotide metabolism. Hierarchical clustering for mutant strains shows similar classification of the aero-type as assigned by their locations on the rate-yield plane. Enrichment of genes in each cluster is indicated on the bottom.

We constructed the single (Δ*ndh*, Δ*nuoB*, Δ*cydB*, and Δ*cyoB*) and double (Δ*ndh*Δ*cydB*, Δ*ndh*Δ*cyoB*, Δ*nuoB*Δ*cydB*, and Δ*nuoB*Δ*cyoB*) knockout strains to test the predicted phenotypic effect experimentally (Materials and methods). Phenotype characterization of the eight mutants qualitatively captured the computationally predicted trends (Fig 3A and S2 Table), and showed that the designed removal of the ETC genes was able to restrain the mutant within the corresponding aero-type at different temperatures (S11 Fig). Additional evidence came from Portnoy et al. [37], where all terminal cytochrome oxidase genes (*cydAB*, *cyoABCD*, and *appBC*) and a quinol monooxygenase gene, *ygiN*, were removed from the *E. coli* genome. This mutant strain was characterized by the lowest possible *Y* and highest *q*_*glc*_, corresponding to aero-type *i* (*f*_*ATPS*_ = 0).

Next, we confirmed the correlation between aero-type and the proteomic state of the mutant strains using RNA-Seq analysis (Materials and methods). Hierarchical clustering of the expression profile showed groupings consistent with the aero-type assigned on the rate-yield plane (Fig 3B). For example, the Δ*cyoB* mutants grouped together in lower aero-type regardless of their large difference in growth rate and glucose uptake rate. Genes involved in central metabolism were also clustered in two main groups (Fig 3B). Consistent with the metabolic state shift shown in Fig 1B, aerobic respiration and metabolic activity decrease, while anaerobic respiration increases as the assigned aero-type goes down from *v* (yellow) to *ii* (purple).

In short, we design mutant strains where the major ETC enzymes are removed combinatorially to perturb the cell’s respiratory potential and ATP production strategy. We show that the phenotypic outcome, proteome re-allocation, and the phenotypic aero-type switch of these strains are consistent with the computational predictions.

### Stratification of the anaerobic phenotypes using nitrate as the electron acceptor

As a facultative anaerobe, *E. coli* is able to thrive under a variety of environmental conditions, from highly oxic to completely anoxic, with its amazingly versatile pool of fifteen primary dehydrogenases and ten terminal reductases [38]. So far, we have discussed how the differential usage of approximately one third of these enzymes gives rise to a stratified phenotypic distribution during aerobic growth when oxygen is used as a terminal electron acceptor. How do optimal phenotypes distribute on the rate-yield plane under anaerobic condition if alternative dehydrogenases and terminal reductases are activated?

To answer this question, we created an in silico strain where the expression of all terminal cytochrome oxidase genes (*cydAB*, *cyoABCD*, and *appBC*) and a quinol monooxygenase gene (*ygiN*) were set to zero. This mutant strain was shown to produce a phenotype that was almost incapable of oxygen utilization and presented fermentative behavior under oxic condition [37]. Considering that nitrate represses other anaerobic pathways in *E. coli* under anoxic conditions [38], we supplemented nitrate to be utilized as the preferred electron acceptor instead of oxygen, performed strain sampling simulations, and examined the fitness distribution.

**Fig 4:**
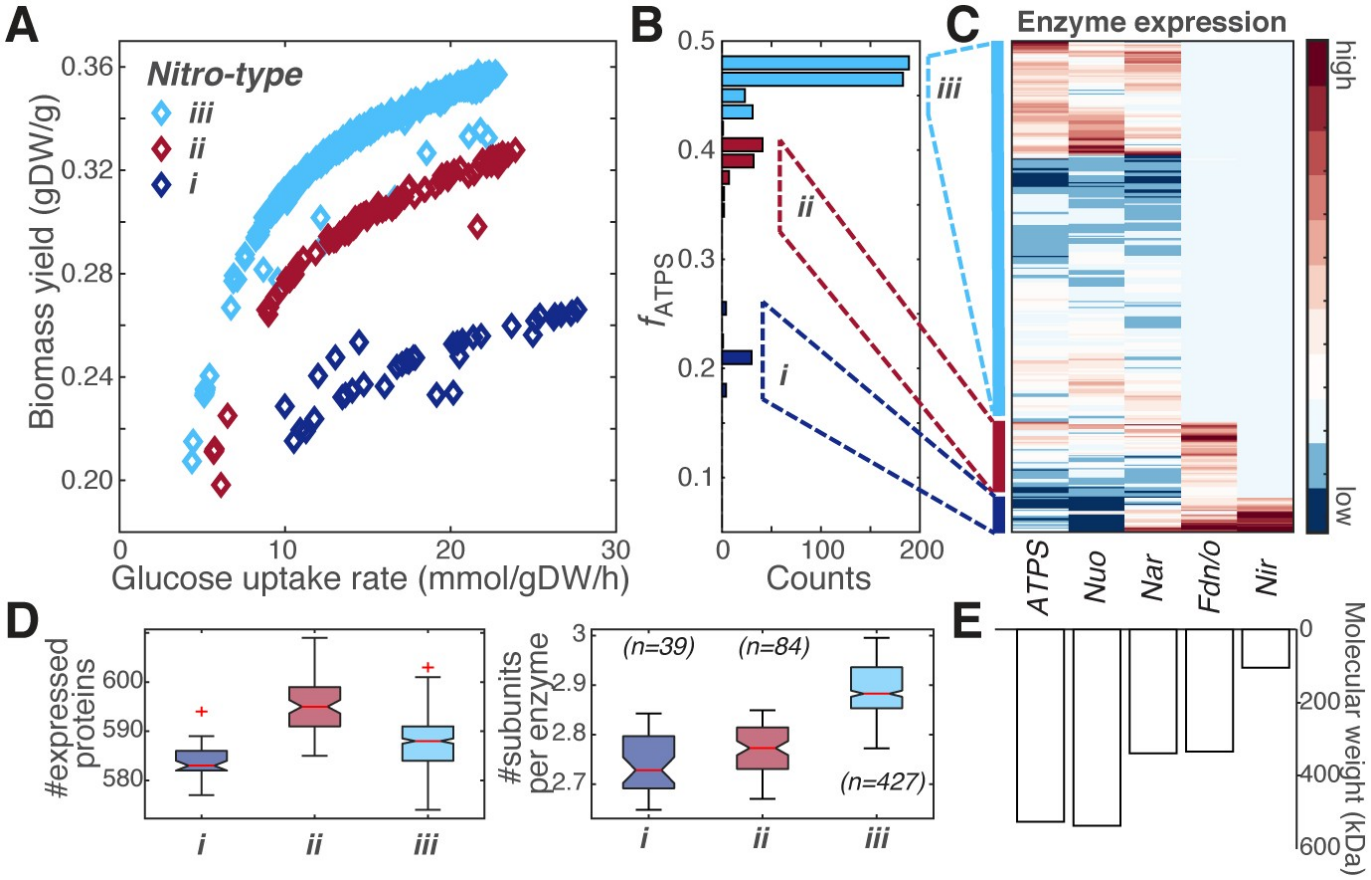
The stratified phenotypic distribution under anaerobic condition supplemented with nitrate. (A) Fitness effect calculated from 550 in silico *E. coli* strains grow anaerobically using nitrate as the electron acceptor. Three anaerobic respiratory states (nitro-type *iii* in cyan, nitro-type *ii* in red, and nitro-type *i* in dark blue) can be clearly identified on the rate-yield plane, which distribute in a stratified fashion similar to the aero-types. (B) Distribution of the computed *f*_*ATPS*_, showing three peaks correlated with the three populated nitro-types on the rate-yield plane shown in panel A. (C) Simulated concentrations of the enzymes involved in oxidative phosphorylation across the 550 sampling simulations. Data shown on the third column labeled with “Nar” is the sum of concentrations for the nitrate reductase A and Z; and on the fourth column labeled with “Fdn/o” is the sum of concentrations for the formate dehydrogenase N and O. (D) Proteome complexity, measured by the calculated number of proteins expressed (left) and the average number of subunits per enzyme (right) in the optimal solution. The data is represented as box plots with the central red line showing the median, the bottom and top edges indicating the 25^*th*^ and 75^*th*^ percentiles, and whiskers extending to 1.5 times the interquartile range. The non-overlapping notches on the boxplot show that the medians between groups differ with a 95% confidence. Number of samples in each box is indicated in parenthesis. (E) Molecular weight of the protein complexes shown in panel C. The molecular weight labeled with “Nar” is the averaged of the nitrate reductase A and Z. The molecular weight labeled with “Fdn/o” is the averaged of the formate dehydrogenase N and O.

Three discrete anaerobic phenotypes were found that distributed in a stratified fashion on the rate-yield plane (Fig 4A). Consistent with the aero-type analysis, each observed phenotype can be characterized by a particular *f*_*ATPS*_ value (Fig 4B), usage of a different combination of the respiratory enzymes (Fig 4C), and different proteome complexity (Fig 4C and 4D). By analogy but not to be confused with the “aero-type” where oxygen is used as the terminal electron acceptor, we denoted these anaerobic phenotypes “nitro-type” *i* ∼ *iii*. Nitro-type *i* with the lowest biomass yield expressed the siroheme NADH-nitrite reductase NirAB in addition to the high expression of the nitrate reductase A or Z. This small-molecular-weight enzyme likely helped to reduce proteome complexity through either detoxifying nitrite generated by the nitrate reductases, or by carrying out fermentative ammonification that balanced between maximizing ATP production and maintaining the NAD^+^ levels [39]. These results again emphasized the importance of proteome allocation to the energy production pathways in determining the phenotypic distribution.

## Discussion

In this study, we develop a systems biology definition of the phenotypic fitness landscape based on the solution space of the *E. coli* genome-scale FoldME model [33]. Simulations that sample thousands of *E. coli* strains across many temperatures lead to the discovery of a stratified geometry of phenotypic distribution, which is consistent with observations from a compendium of experimental phenotypic data. FoldME’s capability to reveal quantitative multi-level relationships between a cell’s genotype, metabolic state, proteomic allocation, energy production strategy, and the phenotype provides us with the opportunity to interpret the observed topography of the phenotypic fitness landscape [19, 40]. We find that: 1) the stratification is due to the discreteness of the ATP production strategy; 2) the fraction of the ATP produced by the ATP synthase (*f*_*ATPS*_) is a governing parameter describing the discretization; and 3) the discretization is rooted in a balance between the modularity of proteome composition and metabolic functions underlying optimal growth.

The direct correlation between a cell’s energy production strategy and the phenotypic landscape topography inspires the definition of the *E. coli* “aero-type” to summarize the complex relationships between genotype, metabolic state, proteome allocation, and phenotype. We reason that a switch in the aero-type may occur if differential usage of the ETC enzymes is imposed by genetic mutations or environmental stresses. To confirm the hypothesis, we experimentally construct the mutant strains where major ETC enzymes are removed combinatorially, and show that the measured aero-types of the mutants are consistent with computational prediction.

With the aero-type defined as a key phenotypic descriptor, it is worth pointing out that discretization of ATP production through other reactions (represented by *f*_*PGK*_, *f*_*PYK*_ and *f*_*ACKr*_, S8(B)–S8(D) Fig) within each aero-type is also observed (S8(B)–S8(D) Fig). Based on these results, we propose a multi-level regulation that the cell uses to adjust its energy production strategy in adaptation to genetic and environmental perturbations (S12(A) Fig). A cell first partitions its cellular resources between the ATP synthase and enzymes that catalyze other ATP-production reactions to meet the minimal ATP requirement for growth. Thus, an aero-type is determined. Next, within each aero-type, two types of reactions further fine-tune the ratio between the proteome dedicated to ATP and biomass precursor production, respectively: 1) those that produce both ATP and biomass precursors, such as PGK and PYK, and 2) ACKr that contributes to ATP production alone. The final result optimizes the ratio between ATP and biomass precursors to maximize biomass production in adaptation to a particular condition that the cell encounters.

This two-level regulation is consistent with the underlying physical principles of the respiration-fermentation tradeoff on the top level, and thermodynamic tradeoff between biomass and ATP yield on the second [41, 42]. Moreover, our formulation offers critical mechanistic details compared to similar efforts that model energy metabolism as a partition between the parallel pathways of the high-yield, low-yield ATP producers and the biomass producer [43, 44], therefore extends the explanatory power beyond the constrained boundaries on the rate-yield plane to the full scope of a fitness landscape.

The proposed hierarchical energy production strategy may find applications in diverse fields such as metabolic biochemistry, cellular physiology, and evolutionary dynamics. For example, rate-yield tradeoff is one of the long-standing questions in understanding bacterial physiology [45], yet controversy as to whether a positive or negative relationship should be seen still exists [46, 47]. On top of what relationship should result, mechanistic interpretations also come in variety of forms: proteome investment tradeoff between the metabolic enzymes and the uptake system of the limiting nutrient [48, 49], efficiency tradeoff between the fermentation and respiration enzymes [50, 51], or tradeoff between membrane efficiency and ATP yield [52], to name a few. Our results help put forward a generalized yet straightforward reconciliation of these different points of view. If the energy production strategy (or aero-type) remains the same, a positive rate-yield correlation should be seen. When the current energy plan is not capable of supporting growth and a switch to another aero-type must occur, phenotypic tradeoffs result.

The phenotypic landscape defined based on aero-type also offers an alternative perspective to understand bacterial adaptation towards optimal fitness. Instead of “climbing up the fitness peak”, mutations that arise during evolution could move the phenotype in two directions: one towards higher growth rate, biomass yield, and nutrient uptake rate where the cells remain in the same aero-type; and the other in an orthogonal direction where an aero-type switch is anticipated under constant growth rate. The fitness effect of a particular mutation can then be analyzed through its influence on the metabolic network and proteome re-allocation, which is governed by the fundamental physicochemical principles regarding fermentation-respiration and thermodynamic tradeoffs. We present an initial attempt to contextualize this perspective on bacterial evolutionary dynamics (S2 Text and S12(B) and S12(C) Fig), and expect subsequent studies to investigate how this framework may help us understand the convergence and divergence, predictability and stochasticity of bacterial evolution.

The concept of a fitness landscape has shaped thinking in evolutionary biology since the 1930s when it was first articulated. Here, we put forward a low-dimensional representation of the fitness landscape by quantifying the metabolic and proteomic state using the relative contributions of a few key ATP-producing reactions. Our analysis suggests that the topology of this fitness landscape is encoded in the energy allocation strategy underlying an organism’s metabolic network and proteome complexity. The influence of environmental fluctuations (e.g., temperature change, the presence and absence of oxygen) and genetic perturbations (e.g., different sampling strategies on enzyme efficiency and protein stability) on the fitness landscape can be rationally evaluated based on how the cell’s energy production is regulated. In principle, such a fitness landscape should be a general and effective framework with which to understand adaptation and evolution of different cell types in a variety of organisms (e.g., Crabtree effect for yeast and Warburg effect for cancer cells) under diverse conditions.

## Materials and methods

### Literature compendium of *E. coli* phenotypes

Rate and yield are the most fundamental quantities used to describe bacterial ecology and physiology. The rate can be measured as growth rate, or moles/grams of substrate, ATP, or biomass production per unit time. Yield is usually measured by moles/grams of biomass or ATP per unit of substrate. Regardless of which definition of rate and yield to use, these two physiological parameters are tightly correlated with each other. However, the exact form of the relationship is context-dependent, which may vary according to different experimental procedures and conditions. Here, we aim to resolve the controversy and provide a unified explanation for the condition-dependent rate-yield correlation. Therefore, the particular definition should not affect our investigation and discussion. Without loss of generality, and to compare with the genome-scale model simulations using glucose as carbon source, we choose to use the substrate (glucose) uptake rate (*q* (*q*_*glc*_), mmol/gDW/h) and the biomass yield (gDW/g) to denote the *E. coli* phenotypic space.

Substrate uptake rate (*q*) and growth rate (*μ*) are collected from two main types of experimental measurements (S1(B) Fig, top left): 1) growth in nutrient chemostat [27, 53–57], and 2) characterization of the ALE end-point strains [32, 34, 35, 37, 58–64]. Biomass yield is then calculated as $\frac{\mu }{q\cdot m}$, where *m* is the molecular mass of the substrate. A total of 199 data points result in the phenotypic space spanned by substrate uptake rate and biomass yield, including measurements taken for wild-type *E. coli* and gene knockout strains (S1(B) Fig, top right), under different nutrient conditions (S1(B) Fig, bottom left), and with different *E. coli* strains (S1(B) Fig, bottom right). Despite the broad difference in data sources, the phenotypic characterization of *E. coli* seems to occupy a common space with an interesting structure that is discussed in the Results.

### An overview of the FoldME model

All sampling simulations in this paper are performed using the recently developed genome-scale model for metabolism and protein expression enhanced with the chaperone network, FoldME [33]. The reconstruction of FoldME started with associating all biochemical reactions in the *E. coli* genome-scale ME-Model *iOL*1650 [31] with the sequences and structures of their catalytic enzymes [65]. Then we computed the temperature-dependent folding properties for every modeled protein, with which the protein’s condition-specific chaperone requirement was formulated. Next, we coupled the folding state of the cell into its metabolic network by allowing three folding pathways (spontaneous, DnaK-assisted, and GroEL/ES-mediated) to compete for folding of any protein based on the calculated chaperone requirement. As such, the model was capable of adjusting the in vivo folding pathway of each protein to minimize the global cost invested in chaperone biosynthesis and the energy requirement for folding.

The choice of parameters is critical for applying genome-scale models to understand biological phenomena on the systems level. The FoldME model is constructed based on three basic categories of parameters: 1) the global physiological parameters, 2) the in vivo turnover rate of metabolic enzymes, and 3) the protein-specific thermodynamic parameters. The first two categories of parameters are common to all ME-models, and thus are set to the default values as first developed in O’Brien et al. [31]. Protein-specific thermodynamic parameters, including the kinetic folding rate, free energy of unfolding, and aggregation propensity, are unique to the FoldME model. These parameters are calculated using protein sequences and structures with empirical prediction algorithms that are well established in literature. More details of model formulation, parameter calculation, and sensitivity analysis can be found in Chen et al. [33].

We showed that the FoldME model improved the precision and scope of prediction for the optimal proteome composition over a wide variety of perturbations, including temperature, nutrient availability, and genetic mutations, and is therefore suitable for the study of phenotypic distribution presented in this paper.

### *E. coli* strain sampling simulations

The purpose of our sampling method is to evaluate the phenotypic distribution of *E. coli* using in silico strains reconstructed to represent the diversity of naturally occurring strains. We assume that adaptation is achieved through gradual accumulation of large amounts of mutations that emerge independently, each with a random small effect on the affected genes. To simulate the genome-scale consequence of this long-term dynamic evolutionary process and estimate its fitness effect, we design a two-step process: 1) select genes for mutation according to the probability of observing a mutation in each gene, and 2) determine the molecular effect of the mutation on the corresponding gene.

In the first step, we analyzed the genetic variations of 1,765 *E. coli* strains collected from 1) the PATRIC database [66], 2) the Ecoref strain panel [67], and 3) a manually curated set of adherent-invasive *E. coli* (AIEC) strains. We compiled protein sequences for the 1,566 protein-coding genes present in the FoldME model, and performed pairwise sequence alignments of the protein from each strain against its homologous sequence in *E. coli* K12 MG1655 [68]. We found a total of 266,940 coding region mutations, including 245,635 non-synonymous SNP, 16,591 deletions and 4,714 insertions. Then we defined the probability of observing a mutation in a gene as the number of all observed mutations in that gene over the total number of coding region mutations. Next, we need to determine which genes harbor mutation in each sample. To do that, we generated a random number between 0 and 1 for each gene. If the random number is smaller than the gene’s mutation frequency, the gene is mutated; otherwise, the gene is left in its wild-type form. As such, we reproduced the probability of observing a mutation in a gene in the naturally occurring *E. coli* strains (S2 Fig).

In the second step, we perturbed the catalytic efficiency and the thermo-stability of the enzymes selected for mutation in the first step. The beneficial and deleterious effects of mutations were known to distribute exponentially, with many small‑effect mutations and fewer large‑effect ones [69, 70]. To reflect the exponential distribution of beneficial effect at the gene level, we scaled the in vivo turn-over rate (*k*_*eff*_) of the affected enzyme with an exponentially distributed random number between 0.5 and 2. In the same time, we perturbed the enzyme’s thermo-stability (free energy of unfolding Δ*G*) by a random amount between -2 to 2 kcal/mol to account for (de)stabilizing mutations with a small effect. The direction of change in the enzyme efficiency and stability was assumed to be opposite considering the pleiotropic effects of mutations [71], such that if the enzyme’s efficiency increased, its stability decreased, and vice versa.

Finally, to introduce environmental stresses, we simulated 100 strain samples at each temperature from 25 to 46°C, resulting in a total of 2,200 simulations.

To test the robustness of this sampling process, we performed additional sets of simulations with the following modifications: 1) fixed the number of mutated genes to 10%, 20%, or 30% of the total number of modeled genes, and selected mutations assuming uniform mutation fixation frequency for all genes; 2) perturbed only the *k*_*eff*_ or the stability of the enzymes selected for mutation; 3) used a different wild-type *k*_*eff*_ profile according to the recent machine learning study [72]; and 4) perturbed the *k*_*eff*_ of the mutated enzyme with larger scaling factors. None of these modifications in the sampling procedure changed our main conclusion regarding a stratified phenotypic landscape determined by the multimodal distribution of *f*_*ATPS*_. As an example, we showed the result for sampling simulations in which *k*_*eff*_ was scaled between 0.1 to 10 fold, and 0.01 to 100 fold (modification #4, S9(C) Fig). In both cases, *f*_*ATPS*_ distributed around the same locations as shown in Fig 1C and S9(B) Fig, with differences only in the relative amplitude of the fitness peaks. Therefore, we considered our current choice of sampling procedure and parameters capable of generating robust phenotypic predictions with evolutionarily meaningful genotypes.

### Sampling simulations with fixed *f*_*ATPS*_

To confirm the relationship between the multimodal distribution of *f*_*ATPS*_ and the stratified structure of the phenotypic fitness landscape, we performed sampling simulations where *f*_*ATPS*_ was constrained to its five most likely values: 0, 0.37, 0.53, 0.64, and 0.71. The constraint was formulated as followed:

$$\begin{array}{c} \left(1-p\right)\cdot V_{ATPS4rpp}=p\cdot \left(V_{PGK}+V_{ACKr}+V_{PYK}+V_{PPKr}+V_{PPK2r}+V_{SUCOAS}+V_{PRPPS}\right) \end{array}$$ (1)

where *V*_*reaction*_*name*_ denoted the flux of the corresponding reaction and *p* was the value that *f*_*ATPS*_ was constrained to. For every *f*_*ATPS*_ value, 24 sampling simulations were performed at each temperature from 25 to 46°C. However, this strong constraint caused many incompatibilities with the sampled genotype, resulting in a final 2,237 feasible and optimal solutions reported in S8 Fig.

### Sampling simulations at fixed growth rate

Difference in growth temperature gave rise to systematic changes in protein stability and in vivo turnover rate of the enzymes, consequently different growth rates [33]. To rule out the possibility that the multimodal distribution of *f*_*ATPS*_ was a result of the bias induced by growth rate difference at different temperatures, we performed additional sampling simulations at one particular temperature. In the same time, it was desirable to cover as much on the rate-yield space as possible. Thus, we examined the accessible range of *q*_*glc*_ and *Y* (i.e., values that render feasible solutions for cell growth) at each temperature in the previously described 2,200 sampling simulations (S9(A) Fig). In general, below the optimal growth temperature, accessible ranges of *q*_*glc*_ and *Y* both decreased as temperature increased, favoring the choice of lower temperature. Then, we considered the overlap with the most populated experimental phenotype range (S1(A) Fig), where *q*_*glc*_ varied approximately in the range between 5 to 15 mmol/gDW/h and *Y* between 0.3 to 0.55 gDW/g. Combining both criteria, we fixed the second set of sampling simulation at 30°C (S9(A) Fig, red).

Next, to maximize instances in every discrete *f*_*ATPS*_ regime and enable direct comparison in metabolic fluxes, we focused sampling along a few *μ*-isoclines. We chose six growth rates (values reported in relative to the WT growth rate at 37°C): 3 around the average growth rate at 30°C (0.36, 0.44, 0.47), one close to the upper limit for growth at 30°C (0.65), and two slightly lower than the lower bound (0.18, 0.22). Simulation at higher fixed growth rate generated large number of infeasible solutions, thus were not included. The results confirmed that at each simulated growth rate, *f*_*ATPS*_ showed similar multiple Gaussian distribution that differed only in the relative weight of each Gaussian. Because of the same number of peaks and the mean values, we reported in S9(B) Fig the collective result for all six growth rates together.

### Fitting the multimodal distribution of *f*_*ATPS*_

We assumed the *f*_*ATPS*_ value for each aero-type to be normally distributed. It followed that *f*_*ATPS*_ calculated from the sampling simulations should be fitted to a mixture of multiple Gaussian distributions, each representing one aero-type. The number of Gaussian distributions (peaks) should be chosen as the number of aero-types determined based on the distinguishable metabolic (Fig 1) and proteomic states (Fig 2). Therefore, we consider *f*_*ATPS*_ = 0 (the fully fermentative phenotype) as one “peak”, and fitted the remaining data with four Gaussians using Matlab.

To check whether our choice for the number of peaks was consistent, we compared distributions generated from many separate sets of sampling simulations, including those using different sampling strategies for sensitivity analysis. The *f*_*ATPS*_ distribution constantly showed five peaks, although the heights of the peaks varied. Peaks around 0.0, 0.37 and 0.53 were clearly present throughout all data sets, whereas peaks around 0.64 and 0.71 could be blurred under certain conditions. This final uncertainty likely came from unresolved proteome complexity of the highly respiratory phenotypes, which should not impair the validity of the fitting and the sampling process.

### Bacterial strains

The *E. coli* electron transport chain contains two types of enzymes: a dehydrogenase that oxidizes an electron donor and a cytochrome oxidase that reduces the electron acceptor (S10(A) Fig). To create mutant strains that are constrained to a particular aero-type, we choose two enzymes from each category to be removed from the genome: NADH dehydrogenase I (NuoABCDEFGHIJKLMN) and NADH dehydronase II (Ndh) for the dehydrogenase; cytochrome *bo* oxidase (CyoABCD) and cytochrome *bd*-I oxidase (CydAB) for the cytochrome oxidase.

Three of the chosen ETC enzymes are multi-protein complexes, and we aim to choose the gene that maximally disrupts the function of the whole enzyme. For NADH dehydrogenase I, all subunits are required for the assembly or stability of a functional enzyme [73]. The subunit encoded by the gene *nuoB* contains the N2 4Fe-4S cluster, which may play a role in proton translocation activity of the enzyme [74]. For cytochrome *bd*-I oxidase, although both subunits are required for binding of the heme *b*_595_ and heme *d* components of cytochrome *bd*-I, subunit II encoded by gene *cydB* binds a structural ubiquinone-8 cofactor that may have a role in the dimer assembly [75]. Similarly, deletion each gene in the *cyo* operon results in nonfunctional enzymes, yet we choose to disrupt *cyoB* because it encodes subunit I which is involved in proton translocation [76].

The four single-ETC-gene-knockout and four double-ETC-gene-knockout strains were constructed with the P1 phage transduction method [77], using *E. coli* K-12 MG1655 (ATCC 700926) as the recipient strain. Keio collection strains were used as donor strain for the generation of gene knockout cassettes containing a kanamycin resistance marker [78]. Knock-outs were confirmed by PCR and DNA resequencing (S3 Table).

### *E. coli* phenotype characterization

Characterizations were performed fully aerated, at 37°C, in 15 mL working volume tubes containing M9 glucose medium, as described in LaCroix et al. [34]. Cultures were initially inoculated from frozen glycerol stocks, and grown overnight. Physiological adaptation was achieved by growing exponentially over 2 passages for 5 to 10 generations. Cultures were then passaged to a fresh tube, and spectrophotometer optical density (OD) readings were periodically taken at a wavelength of 600 nanometers (Thermo Fisher Scientific, Waltham, MA) until stationary phase was reached.

Samples were filtered through a 0.22 micrometer filter (MilliporeSigma, Burlington, MA) at the same time OD measurements were taken, and the filtrate was analyzed for glucose concentrations using a high-performance liquid chromatography system (Agilent Technologies, Santa Clara, CA) with an Aminex HPX-87H column (Bio-Rad Laboratories, Hercules, CA). Glucose uptake rates in exponential growth were determined by best-fit linear regression of glucose concentrations versus cell dry weights, multiplied by growth rates over the same sample range.

The oxygen uptake rate of each aerobic culture was determined by measuring the rate of dissolved oxygen depletion in an enclosed respirometer chamber using YSI 5300A Biological Oxygen Monitor System that utilized Clark type polarographic oxygen probes (Cole-Parmer Instruments, Vernon Hills, IL).

### DNA resequencing

To determine the mutations emerged during adaptive laboratory evolution of the *pgi*-deficient *E. coli* strain, growth-improved clones along the ALE trajectory were isolated and grown in M9 minimal medium supplemented with 4g/L glucose. Cells were then harvested while in exponential growth and genomic DNA was extracted using a KingFisher Flex Purification system previously validated for the high throughput platform mentioned below [79]. Shotgun sequencing libraries were prepared using a miniaturized version of the Kapa HyperPlus Illumina-compatible library prep kit (Kapa Biosystems). DNA extracts were normalized to 5 ng total input per sample using an Echo 550 acoustic liquid handling robot (Labcyte Inc), and 1/10 scale enzymatic fragmentation, end-repair, and adapter-ligation reactions carried out using a Mosquito HTS liquid-handling robot (TTP Labtech Inc). Sequencing adapters were based on the iTru proptocol [80], in which short universal adapter stubs were ligated first and then sample-specific barcoded sequences added in a subsequent PCR step. Amplified and barcoded libraries were then quantified using a PicoGreen assay and pooled in approximately equimolar ratios before being sequenced on an Illumina HiSeq 4000 instrument.

### RNA-Seq data acquisition and analysis

Total RNA was sampled from duplicate cultures. All strains were grown in M9 minimal medium supplemented with 4g/L glucose. 3 mL of cell broth (taken at OD600 ∼ 0.6) was immediately added to 2 volumes Qiagen RNAprotect Bacteria Reagent (6 mL), vortexed for 5 seconds, incubated at room temperature for 5 min, and immediately centrifuged for 10 min at 17,500 RPMs. The supernatant was decanted, and the cell pellet was stored at -80°C. Cell pellets were thawed and incubated with Readylyse Lysozyme, SuperaseIn, Protease K, and 20% SDS for 20 minutes at 37°C. Total RNA was isolated and purified using the RNeasy Plus Mini Kit (Qiagen) columns following vendor procedures. An on-column DNase treatment was performed for 30 minutes at room temperature. RNA was quantified using a spectrophotometer (NanoDrop 1000, Thermo Fisher Scientific, Waltham MA) and quality was assessed by running RNA electrophoresis on the Agilent 2100 Bioanalyzer (Agilent Technologies, Santa Clara CA). The rRNA was removed using Illumina Ribo-Zero rRNA Removal Kit (Gram-Negative Bacteria). Stranded RNA-Seq Kit (Kapa Biosystems) was used following the manufacturer’s protocol to create sequencing libraries with an average insert length of around ∼300 bp. Libraries were sequenced on an Illumina HiSeq 4000 instrument.

Raw sequencing reads were obtained as described above, and mapped to the reference genome NC_000913.3 using Bowtie 2.3.4.3 [81] with the following options “-X 1000 -N 1 -3 3”. Transcript abundance was quantified using summarizeOverlaps from the R GenomicAlignments package, with the following options “mode=“IntersectionStrict”, singleEnd = FALSE, ignore.strand = FALSE, preprocess.reads = invertStrand” [82]. Transcripts per Million (TPM) was calculated by DESeq2, and log-transformed TPM (*log*_2_(*TPM*+ 1)), referred to as log-TPM, was taken for the downstream analysis. The log-TPM values of the two biological replicates were highly correlated (*R*^2^ > 0.97), expect for the Δ*ndh*Δ*cydB* mutant (*R*^2^ = 0.91). Uncertainty of the Δ*ndh*Δ*cydB* mutant might come from partial knockout for one of the replicates, which showed relatively high expression of the *cydB* gene. We considered the aero-type assignment and other quantifications for this mutant to be less reliable compared to others.

Principal component analysis (PCA) of the log-TPM showed that the first four components could explain 84% of the variations throughout the expression profile. The first principal component was highly correlated with exchange rates such as the glucose/oxygen uptake rate and the acetate production rate, and the second with growth rate. These components, although highly explanatory, were enriched in gene clusters (e.g., chemotaxis, flagellum biosynthesis, amino acid metabolism, sugar transport, etc.) that were non-specific to the conditions and phenotypes that we were interested in. Alternatively, the fourth component, explaining 5.4% of the overall variation, was highly enriched in genes involved TCA cycle, anaerobic respiration and ETC activity. Consequently, we considered selecting genes most representative for aero-type for subsequent hierarchical clustering analysis. First, we calculated the Spearman correlation between five phenotypic parameters (*μ*,*Y* ,*q*_*glc*_,$q_{O_{2}}$, *q*_*ac*_) and our aero-type definition in the sampling simulations. It turned out that only *q*_*ac*_(Spearman correlation = -0.9; P-value = 4e-143) and *Y* (Spearman correlation = 0.87; P-value = 4e-120) showed significant correlation. Then, we computed the Pearson correlation between log-TPM and experimentally measured phenotypic parameters, and selected genes that were highly correlated with *q*_*ac*_ and *Y* (P-value<0.01), but not with *μ*. This process resulted in a set of 391 genes, which were used for generating the clustering diagram shown in Fig 3B. As expected, this set was enriched in genes involved in oxidative phosphorylation (17 out of 94, one-sided binomial test P-value = 0.004) and TCA cycle (7 out of 27, one-sided binomial test P-value = 0.009). The clustering pattern qualitatively resembled that generated using all genes in the expression profile (4314 genes in total), yet it maximized signal of interest for easy analysis and interpretation.

## Supporting information

S1 Text Comparison between “aero-type” and P/O ratio as phenotypic descriptors for *E. coli*. (PDF)

S2 Text Dynamics of bacterial adaptive evolution on the stratified phenotypic landscape. (PDF)

S1 Fig Phenotypic data of *E. coli* measured in experiments. (A) Visualization of a compendium of 199 experimental measurements. Contours of the phenotype density are overlaid on top of the experimental data (gray circles). (B) Experimental phenotypic distribution visualized by whether measurements are taken for WT or evolved *E. coli* strains (top left), for WT or strains with gene knockout (top right), under different nutrient conditions (bottom left), and for different *E. coli* strains (bottom right).
(TIF)

S2 Fig Probability of observing a mutation in a gene in the naturally occurring *E. coli* strains is captured in the sampling simulations. Comparison between the distributions of the number of observed mutations per gene for the 1,765 naturally occurring *E. coli* strains (left) and frequency of mutations per gene in the 2,200 sampling simulations (right).
(TIF)

S3 Fig Principal component analysis for forty-three reactions in central metabolism depicted in Fig 1B. The figures illustrate the observed correlation between phenotypic state and the metabolic fluxes, yet they can be best understood with the definition of “aero-type” introduced in later sections. (A) The metabolic states of the five aero-types can be clearly separated by the first principal component (PC1), representing 61.1% of the data variations. PC2 further decomposes metabolic states into sub-types. (B) PC1 is the only component that’s correlated (Pearson correlation = 0.97) with the flux through ATP synthase (ATPS4rpp, shown in red). Therefore data variations contained in PC1 best represent the observed differences in metabolic states between different aero-types. For example, the fluxes through the TCA cycle are positively correlated with PC1, hence the flux through ATP synthase. This correlation indicates that as the biomass yield (*Y*) decreases along the *μ*-isocline (aero-type decreases from *v* to *i*), flux through TCA cycle also decreases. Similarly, fluxes through acetate overflow are negatively correlated with PC1, hence as *Y* decreases, flux through acetate overflow increases. The opposite sign of correlation between fluxes through the two branches of glycolysis pathways nicely captures the trend that the glycolysis flux slowly switches from oxPPP to EMP as aero-type decreases from *v* to *i*. PC2 (and the principal components thereafter) are not correlated with the flux through ATP synthase, and are not discussed in further details for the purpose of this paper. The name of the reactions are standard reaction IDs available for search on the BIGG database (http://bigg.ucsd.edu/). oxPPP: oxidative pentose phosphate pathway; EMP: Embden–Meyerhof–Parnas pathway; ED: Entner-Doudoroff pathway.
(TIF)

S4 Fig Experimental evidence for the correlation between biomass yield and pathway usage. (A) Phenotypic data of 20 *E. coli* strains under glucose minimal medium are highlighted on the rate-yield space. Data includes two wild-type strains at 37°C (blue circle, labeled with “wt”), one wild-type strain at 42°C (red circle, labeled with “wt”), 7 strains evolved at 37°C (blue circles, labeled with “ale” and the strain number), and 10 strains evolved at 42°C (red circles, labeled with “ale” and the strain number). (B) Mass fraction of the representative pathway is calculated using all genes involved in the corresponding pathway. The Pearson correlation between the biomass yield and the mass fraction of each pathway is shown. Usage of three pathways that are related to aerobic respiration is significantly correlated with biomass yield (shown in bold, with their P-values listed). (C) Biomass yield plotted against the mass fraction of genes involved in oxidative phosphorylation. Each point corresponds to a strain, labeled as in (A). (D) Biomass yield plotted against the mass fraction of genes involved in TCA cycle.
(TIF)

S5 Fig The ATP production reactions and their contribution to phenotypic distance estimated by sampling simulations. (A) Detailed information for the ATP-producing reactions in *E. coli*. Short and full name for the metabolites are listed as follows. 13dpg: 3-Phospho-D-glyceroyl phosphate; 3pg: 3-Phospho-D-glycerate; actp: acetyl phosphate; pyr: pyruvate; pep: phosphoenolpyruvate; succoa: succinyl-CoA; coa: coenzyme-A; prpp: 5-phospho-alpha-D-ribose 1-diphosphate; r5p: alpha-D-ribose 5-phosphate. The fraction of total ATP produced by each reaction varies significantly. The range of variation in the sampling simulations is indicated in the “Fraction” column. (B) Fraction of variations in the rate-yield phenotypic space explained by the indicated ATP-production reactions. 89.5% of the variations in phenotypic distance can be explained by the first six ATP-producing reactions. Among them, oxidative phosphorylation reactions ATPS4rpp and PPKr contributed the most. (C) Comparison between the actual phenotypic distance on the rate-yield plane and that reconstructed from the six ATP-producing reactions. A simulated phenotype is determined by a four-element vector containing the glucose uptake rate, acetate production rate, growth rate and biomass yield. Other typical phenotypic measurements are highly correlated with one or more chosen quantities, and are thus not included in the calculation. Then phenotypic distance is calculated as the Euclidean distance of this vector with respect to that of the wild-type solution at 37°C. Predictors of the stepwise linear regression are taken as the fraction of ATP produced by each reaction listed in (A).
(TIF)

S6 Fig Linear relationship between *f*_*ATPS*_ and the rate-yield phenotype at each specific growth rate. (A) Fitness distribution of the 2,200 simulated strains on the rate-yield plane (same as shown in Fig 1A). Simulated strains along six *μ*-isoclines, which are used in the subsequent analysis shown in panel B, are highlighted. Growth rates shown on the *μ*-isoclines are computed relative to the wild-type growth rate calculated at 37°C. (B) Along each *μ*-isocline, the calculated fraction of total cellular ATP produced by ATP synthase (*f*_*ATPS*_) is linearly correlated with biomass yield (*Y*, top) and glucose uptake rate (*q*_*glc*_, bottom), with a positive and negative slope, respectively. The intercepts of these linear fitting at the minimum and maximum values of *f*_*ATPS*_ (0 and 0.83, respectively) provide a way to estimate the feasible range of *q*_*glc*_ and *Y*. The estimations at each growth rate can be connected to draw the boundary of the rate-yield plane (gray shaded area in panel A). The accessible range of the phenotypic space generated this way encompasses the majority of data points from both experiments and model simulations.
(TIF)

S7 Fig ATP production through the ATP synthase is determined by the aero-type. (A) ATP yield through the ATP synthase is positively correlated with *f*_*ATPS*_ (hence positively correlated with the aero-type as defined in the later Results sections), but not with the mass fraction of the ATP synthase in the proteome. (B) Expression of the ATP synthase is a function of the growth rate and temperature.
(TIF)

S8 FigFiner structure on the fitness landscape.(A) Topography of the fitness landscape reconstructed from constrained sampling simulations at 5 most likely *f*_*ATPS*_ values: 0, 0.37, 0.53, 0.64 and 0.71. (B) Once *f*_*ATPS*_ is determined in the energy production strategy, finer structures can be seen on the phenotypic landscape. The distributions of *f*_*PGK*_, *f*_*ACKr*_, and *f*_*PYK*_ at each fixed *f*_*ATPS*_ value also show distinct multimodal distributions. The pie charts show the allowable energy production strategy that represents over 95% of the solutions at each fixed *f*_*ATPS*_, all fractions shown have standard deviations smaller than 0.02. (C) At fixed *f*_*ATPS*_, biomass yield is negatively correlated with the ratio *f*_*ACKr*_/*f*_*PGK*_. Because acetate is secreted through the ACKr flux and no biomass is made, increase in *f*_*ACKr*_ reduces biomass yield. (D) At fixed *f*_*ATPS*_, the overall ATP yield is positively correlated with the ratio (*f*_*ACKr*_ + *f*_*PYK*_)/*f*_*PGK*_. This ratio reflects the relative efficiency of all ATP-producing reactions in terms of ATP production per unit of substrate. The more inefficient reaction PGK is used (2 ATP per glucose uptake, which is reflected in the value of the fitting curve at $\frac{\left(f_{ACKr}+f_{PYK}\right)}{f_{PGK}}=0$ and *f*_*ATPS*_ = 0), the lower the overall ATP yield is. As the yield of oxidative phosphorylation is much higher (∼34 ATP per glucose), the overall ATP yield increases with *f*_*ATPS*_ at fixed (*f*_*ACKr*_ + *f*_*PYK*_)/*f*_*PGK*_ ratio.(TIF)

S9 Fig Consistency in the *f*_*ATPS*_ distribution from different sets of strain sampling simulations. (A) The phenoptypic variations at each temperature. On this plot, the average values of *Y* and *q*_*glc*_ calculated for the sampled strains at each temperature are denoted by a circle, then the range of accessible values indicated by horizontal and vertical lines going through the average. For T = 25°C, 30°C, 37°C and 40°C, the optimal wild-type phenotype (square) is shown for reference. For T = 25°C, 30°C, and 40°C, shift in the preferred aero-type is shown by the difference in *f*_*ATPS*_ distribution. Eight *μ*-isoclines are drawn, each labeled with the relative growth rate with respect to the simulated optimal WT growth rate at 37°C. Distribution of *f*_*ATPS*_ at 30°C best captures the features of the full distribution, thus we select this temperature for the down-stream analysis. (B) The *f*_*ATPS*_ distribution of the 368 sampling simulations performed at 30°C and selected growth rate. Fitting to a mixture of four Gaussian distributions shows consistency with the observed stratified distribution shown in Fig 1C. (C) *f*_*ATPS*_ value shows a similar multi-modal distribution as the maximum fold change in enzyme efficiency increases to 10 and 100 fold in the sampling simulation.
(TIF)

S10 Fig Comparison of the computed *E. coli* aero-type and P/O ratio. (A) Major protein complexes involved in quinone turnover in the sampling simulations. Formate dehydrogenase N and O catalyze the same reaction, hence are designated to the same complex (FDN/O) for simplicity. Box plot of the normalized expression for the indicated protein complexes shows differential usage of the ETC enzyme between different aero-types. To enable direct comparison, the calculated mass fraction of the enzyme complexes is normalized by the total mass fraction of all ribosomal proteins to remove bias coming from different growth rates. The central red line of the box plot shows the median, the bottom and top edges indicate the 25^*th*^ and 75^*th*^ percentiles, and whiskers extend to 1.5 times the interquartile range. Sample size in each aero-type is the same as in Fig 3D. (B) The activated ETC reactions in the 368 sampling simulations are shown with their relative contributions to the quinone reduction flux and quinol oxidation flux. The calculated biomass yield and acetate production rate are shown to the right, to represent the corresponding simulated phenotype. (C) *f*_*ATPS*_ and the P/O ratio are tentatively binned into five separate groups based on their multimodal distribution, and mapped to the optimal solutions shown in panel B. (D) Comparison of the experimental and simulated relative abundances of selected genes (*ndh*, *cyoB*) with respect to the ATP synthase. Length of the bar and error bar represent the average ratio and standard deviation for each aero-type as defined in Fig 4B for experiment, and in panel C for simulations.
(TIF)

S11 Fig Growth characterizations for the ETC knock-out strains at three different temperatures. The Δ*ndh*Δ*cydB*, Δ*cydB* mutants were chosen to represent the higher aero-types *v*/*iv*, and the Δ*nuoB*Δ*cyoB* mutant was chosen to represent a lower aero-type *ii*. Growth data at 30°C and 37°C nicely recapitulates the expected trend such that Δ*ndh*Δ*cydB* and Δ*cydB* stay in the region for aero-type *iv* and Δ*nuoB*Δ*cyoB* in the region for aero-type *ii*. At 42°C, all three strains generate a lower biomass due to the temperature stress. However, they maintain well separated on the rate-yield plane representing the aero-type constraints caused by the removal of the respective ETC genes. Thus, the presented data supports the notion that the differential usage of the ETC genes determines the phenotypic aero-type of a cell.
(TIF)

S12 Fig Phenotypic outcomes of an ALE experiment on the stratified fitness landscape. (A) The schematic of the proposed hierarchical energy production strategy. Blue and red arrows correspond to the thermodynamic and respiration-fermentation tradeoff, respectively. (B) A coarse-grained representation of the fitness landscape on the rate-yield plane. Color gradient indicates the level of proteome complexity, where blue represents the simpler proteome and red is the more complex proteome. (C) An example adaptive trajectory during the evolution of a *pgi*-deficient strain. (D) Intermediate evolutionary states were chosen at the indicated stages and characterized on the rate-yield plane. Four distinct genotypes were identified along the adaptive trajectory, indicated by red, blue, yellow, and green circles, respectively. Error bars indicate standard deviation of the biological duplicates.
(TIF)

S1 Table Protein complexity for selected metabolic pathways. (PDF)

S2 Table Phenotype comparison of the ETC knock-out strains. (PDF)

S3 Table Sequence of the confirmation primers. (PDF)
