# Supplementary material for: Bacterial fitness landscapes stratify based on proteome allocation associated with discrete aero-types
Source: PLoS Comput Biol. 2021 Jan 19;17(1):e1008596. doi: 10.1371/journal.pcbi.1008596 (PMC7846111; doi:10.1371/journal.pcbi.1008596)
Supplement: S1 Text — (PDF) [file pcbi.1008596.s001.pdf]

S1 Text: Comparison between "aero-type" and P/O ratio as phenotypic descriptors for *E. coli*. The importance of f_{ATPS} and ETC enzyme usage in determining phenotype distribution leads to a computational evaluation for a similar core metabolic parameter, the P/O ratio. P/O is the number of ATP molecules synthesized by oxidative phosphorylation for each pair of electrons passing from a substrate (typically NADH) to O_2. It is fundamental for understanding the cell's energy production efficiency. However, due to complexity of the metabolic network and difficulty of experimental measurement, the exact number for P/O has been under debate for decades. Therefore, the P/O ratio is rarely adopted for practical uses such as comparison between different cell states across conditions and organisms. Here, we compare our definition of "aero-type" with P/O ratio, and discuss their capabilities to describe the respiration behavior and phenotypic potential of an *E. coli* cell.

We calculated the P/O ratio and f_{ATPS} in the 368 sampling simulations constrained at constant growth rates. The result showed that P/O ratio displayed a similar distribution that can be discretized into five categories (S10C Fig bottom). To explore the differences between aero-type and the P/O ratio, we identified the major enzymes involved in the turnover of the cellular quinone pool (S10A Fig), computed the quinone/quinol flux through each enzyme, clustered the simulations according to the flux distribution (S10B Fig), and mapped the corresponding metabolic and phenotypic parameters, including biomass yield and acetate production rate (S10B Fig), aero-type, and P/O category (S10C Fig) to these clusters. The results show that P/O category is solely determined by which combination of ETC enzymes are activated, yet the aero-type tracks the detail structure of the oxidative phosphorylation pathways (relative flux through alternative pathways and additional quinol contribution from substrates other than NADH). For example, when three enzymes, Ndh, FdnGHI/FdoGHI (FDN/O) and CyoABCD (CYO) dominate the oxidative phosphorylation process, the P/O ratio is calculated to be a constant. Yet, as the relative flux between Ndh and FDN/O shift, the aero-type can vary from *ii* to *v*. The distinct aero-types nicely represent the variations in quinol contributions from the TCA enzymes SdhABCD (SDH) and Mqo, and also follow the fluctuations in the overall biomass yield and acetate production rate.

Based on the aero-type assignment for the ETC knockout mutants using their expression profiles (Fig 3B), we propose that aero-type can be determined experimentally by comparing the measured and calculated relative abundance of a few selected genes with respect to the ATP synthase (ATPS). Here, the total abundance of ATP synthase is used as the reference to minimize the bias stems from growth rate and nutrient uptake rate. According to S10D Fig, aero-type *v* is characterized by a low Ndh/ATPS ratio and high CyoB/ATPS ratios; aero-type *ii* behaves the opposite. Such prediction could be further elaborated if more proteomic data enables a thorough evaluation and careful selection of a larger set of representative marker proteins.

In summary, compared to the P/O ratio that outlines the local stoichiometry of the oxidative phosphorylation pathways, aero-type defined by f_{ATPS} is capable of representing the metabolic distribution, phenotypic state, and the associated proteome complexity with a specific energy production scheme. Furthermore, aero-type can potentially be experimentally determined by the relative abundance of a few carefully selected proteins.
