# Supplementary material for: Bacterial fitness landscapes stratify based on proteome allocation associated with discrete aero-types
Source: PLoS Comput Biol. 2021 Jan 19;17(1):e1008596. doi: 10.1371/journal.pcbi.1008596 (PMC7846111; doi:10.1371/journal.pcbi.1008596)
Supplement: S2 Text — (PDF) [file pcbi.1008596.s002.pdf]

S2 Text: Dynamics of bacterial adaptive evolution on the stratified phenotypic landscape. With the definition of aero-type, we review the *E. coli* phenotypic distribution and propose how adaptation towards optimal fitness may be achieved in two directions on the rate-yield plane (S12B Fig). First, bacteria may gain fitness in the direction towards higher growth rate, biomass yield and nutrient uptake rate, where the cells remain in the same aero-type. We reason that mutations that improve the efficiency of a subset of metabolic reactions are likely involved in this process. Adaptation in this direction is usually accompanied by a reduced proteome complexity, yet the magnitude of fitness increase can be restricted by the thermodynamic tradeoff to maintain the free energy difference between substrate and product of the ATP producing reactions (1, 2). When a limit is hit, the cell may seek alternative evolutionary paths in the orthogonal direction where growth rate is constant. A switch in the aero-type is anticipated. Improvement in fitness may be small, but a discrete shift of the metabolic state and re-allocation of the proteome under control of the respiration-fermentation tradeoff are necessary. Mutations with a global regulatory effect on the proteome complexity are considered relevant candidates to achieve this goal. As successive mutations fix, growth rate and aero-type together offer a comprehensive description of the evolutionary path a bacterial strain takes on the rate-yield plane in adaptation to a new environment or genetic perturbation.

To test this hypothesis of evolutionary dynamics, we introduced a dysfunctional gene in central metabolism into the *E. coli* genome, evolved the mutant strain and tracked how the cells adapted to this new condition over time. We chose glucose-6-phosphate isomerase (*pgi*), which is a nonessential gene in the glycolysis pathway that may change the complexity of proteome allocation, and thus perturb the balance of thermodynamic and/or respiration-fermentation tradeoffs. We swapped the native *pgi* with *pgi* from *Brucella melitensis* using a modified gene gorging protocol (3, 4), and evolved the mutant strain under glucose minimal medium for >1,000 generations (4). The characterization of the distinct genotypes isolated from the trajectory showed three jumps on the phenotypic space consistent with the thermodynamic and respiration-fermentation tradeoffs (S12C,D Fig). Whole genome re-sequencing of these clones revealed that the first two jumps were caused by mutations in the *B. melitensis pgi* that restored enzymatic activity, while the last originated from a change in the RNA polymerase β-subunit, which presumably affects global proteome allocation through transcriptional regulation (5, 6).

These dynamics imply that the degree of divergence between evolutionary end points can be affected by the frequency of mutations emerged to modulate either thermodynamic or respiration-fermentation tradeoffs. Each mutation-mediated-tradeoff re-balances the energy production strategy against proteome requirement according to the new condition. The more of these events that occur over the course of adaptation, the more probable it is that evolutionary end points may spread out on the phenotypic fitness landscape.

References

1. Pfeiffer T, Schuster S, Bonhoeffer S. Cooperation and competition in the evolution of ATP-producing pathways. Science. 2001;292(5516):504–507.
2. Pfeiffer T, Bonhoeffer S. Evolutionary consequences of tradeoffs between yield and rate of ATP production. Z Phys Chem. 2002;216(1):51–63.
3. Herring CD, Glasner JD, Blattner FR. Gene replacement without selection: regulated suppression of amber mutations in Escherichia coli. Gene. 2003;311:153–163.
4. Sandberg TE, Szubin R, Phaneuf PV, Palsson BO. Synthetic cross-phyla gene replacement and evolutionary assimilation of major enzymes. Nat Ecol Evol. 2020; p. 1–8.
5. Utrilla J, O'Brien EJ, Chen K, McCloskey D, Cheung J, Wang H, et al. Global rebalancing of cellular resources by pleiotropic point mutations illustrates a multi-scale mechanism of adaptive evolution. Cell Syst. 2016;2(4):260–271.
6. Conrad TM, Frazier M, Joyce AR, Cho BK, Knight EM, Lewis NE, et al. RNA polymerase mutants found through adaptive evolution reprogram Escherichia coli for optimal growth in minimal media. Proc Natl Acad Sci USA. 2010;107(47):20500–20505.
