# Supplementary figures and images for: Bacterial fitness landscapes stratify based on proteome allocation associated with discrete aero-types
Source: PLoS Comput Biol. 2021 Jan 19;17(1):e1008596. doi: 10.1371/journal.pcbi.1008596 (PMC7846111; doi:10.1371/journal.pcbi.1008596)

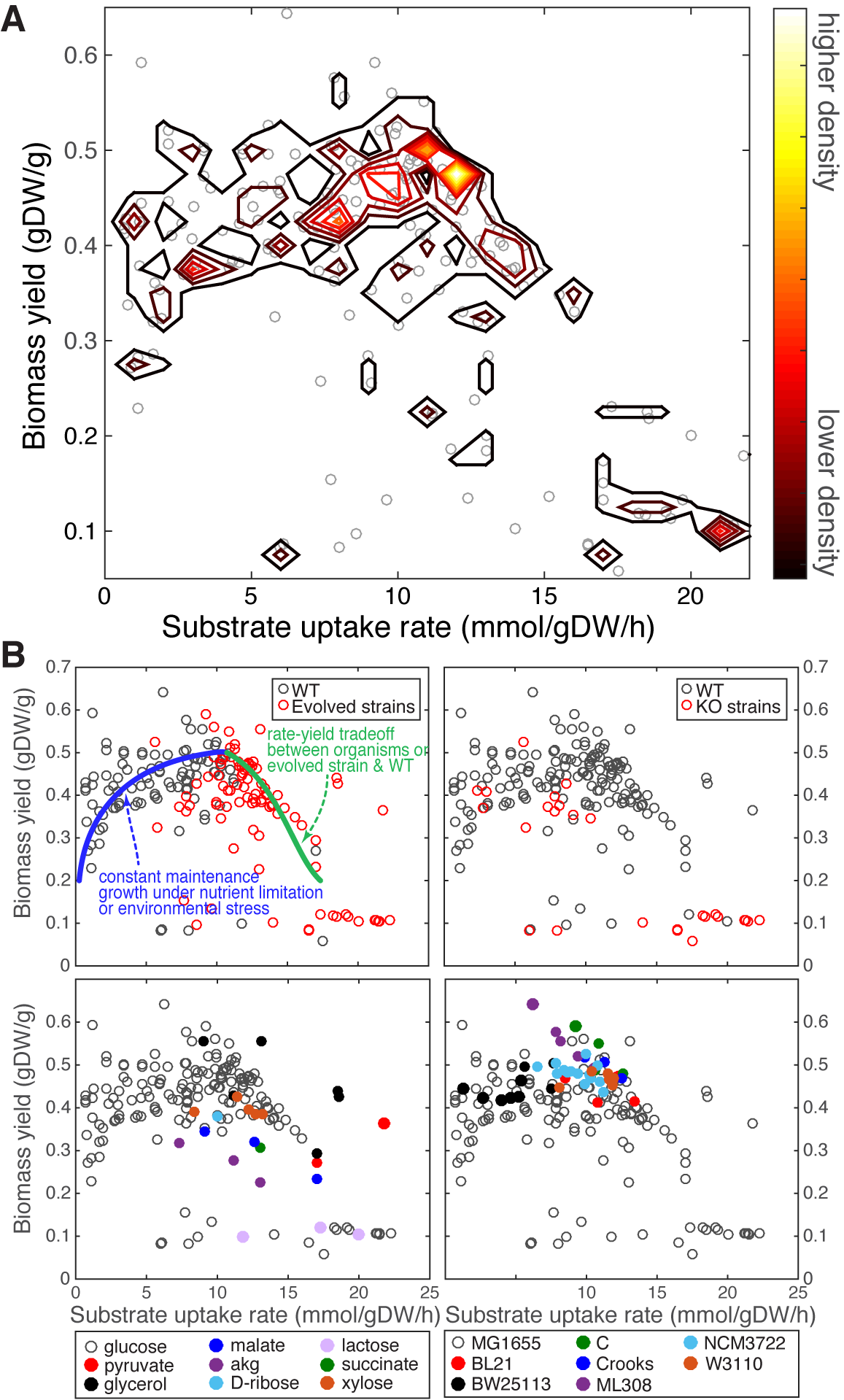

Supplement: S1 Fig — (A) Visualization of a compendium of 199 experimental measurements. Contours of the phenotype density are overlaid on top of the experimental data (gray circles). (B) Experimental phenotypic distribution visualized by whether measurements are taken for WT or evolved E. coli strains (top left), for WT or strains with gene knockout (top right), under different nutrient conditions (bottom left), and for different E. coli strains (bottom right). (TIF) [file pcbi.1008596.s003.tif]

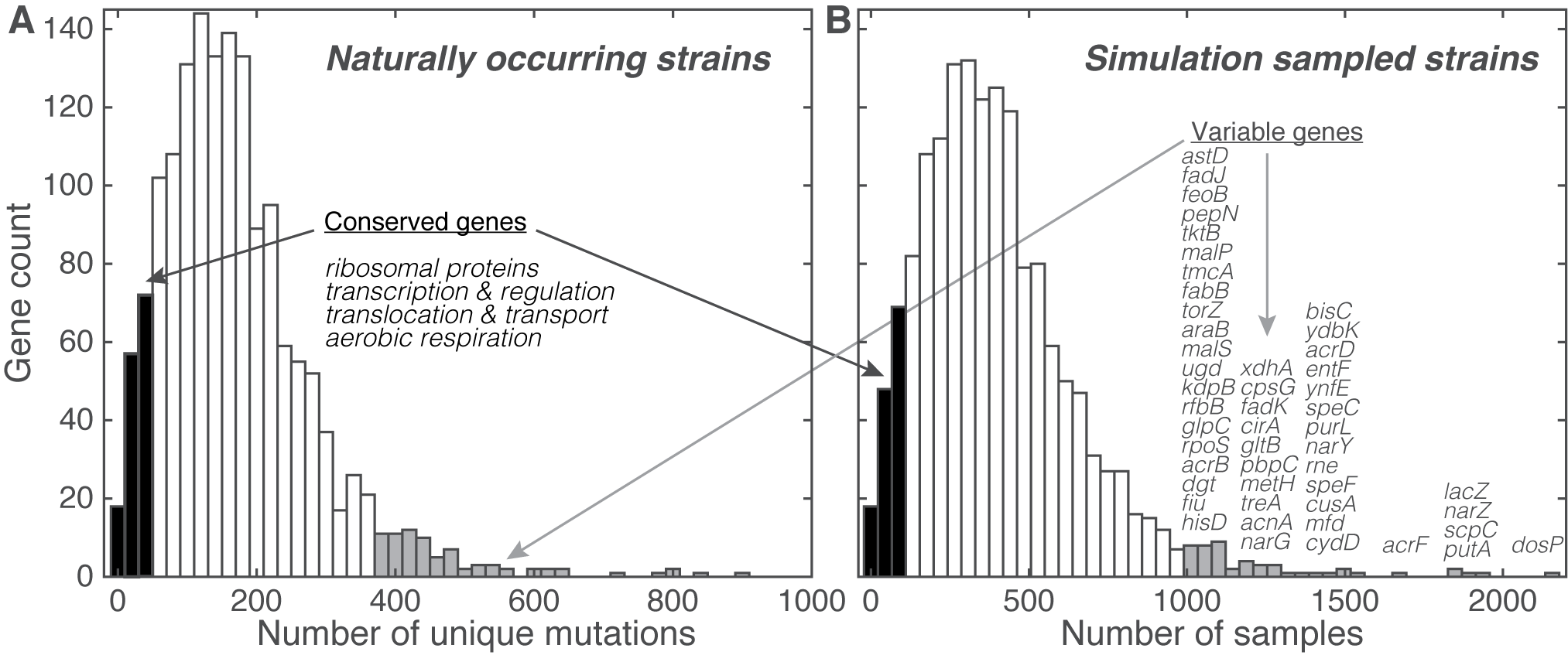

Supplement: S2 Fig — Comparison between the distributions of the number of observed mutations per gene for the 1,765 naturally occurring E. coli strains (left) and frequency of mutations per gene in the 2,200 sampling simulations (right). (TIF) [file pcbi.1008596.s004.tif]

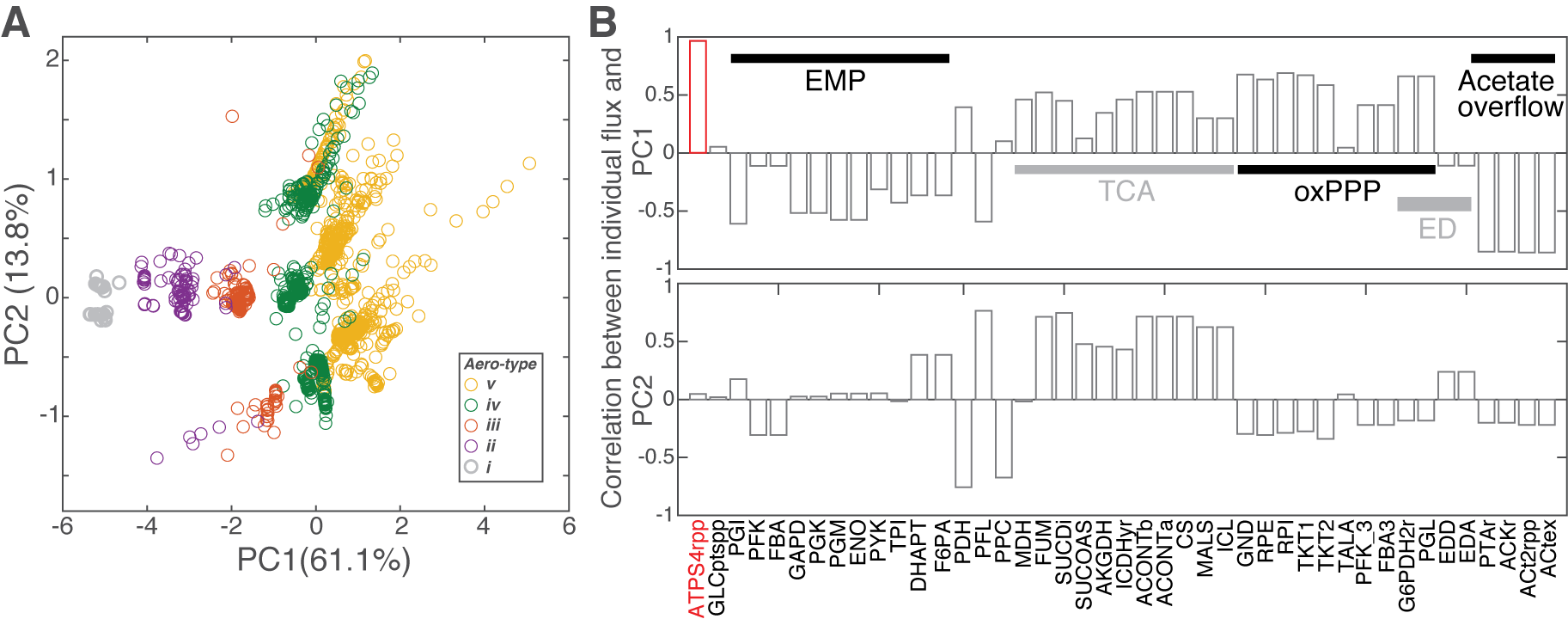

Supplement: S3 Fig — The figures illustrate the observed correlation between phenotypic state and the metabolic fluxes, yet they can be best understood with the definition of “aero-type” introduced in later sections. (A) The metabolic states of the five aero-types can be clearly separated by the first principal component (PC1), representing 61.1% of the data variations. PC2 further decomposes metabolic states into sub-types. (B) PC1 is the only component that’s correlated (Pearson correlation = 0.97) with the flux through ATP synthase (ATPS4rpp, shown in red). Therefore data variations contained in PC1 best represent the observed differences in metabolic states between different aero-types. For example, the fluxes through the TCA cycle are positively correlated with PC1, hence the flux through ATP synthase. This correlation indicates that as the biomass yield (Y) decreases along the μ-isocline (aero-type decreases from v to i), flux through TCA cycle also decreases. Similarly, fluxes through acetate overflow are negatively correlated with PC1, hence as Y decreases, flux through acetate overflow increases. The opposite sign of correlation between fluxes through the two branches of glycolysis pathways nicely captures the trend that the glycolysis flux slowly switches from oxPPP to EMP as aero-type decreases from v to i. PC2 (and the principal components thereafter) are not correlated with the flux through ATP synthase, and are not discussed in further details for the purpose of this paper. The name of the reactions are standard reaction IDs available for search on the BIGG database (http://bigg.ucsd.edu/). oxPPP: oxidative pentose phosphate pathway; EMP: Embden–Meyerhof–Parnas pathway; ED: Entner-Doudoroff pathway. (TIF) [file pcbi.1008596.s005.tif]

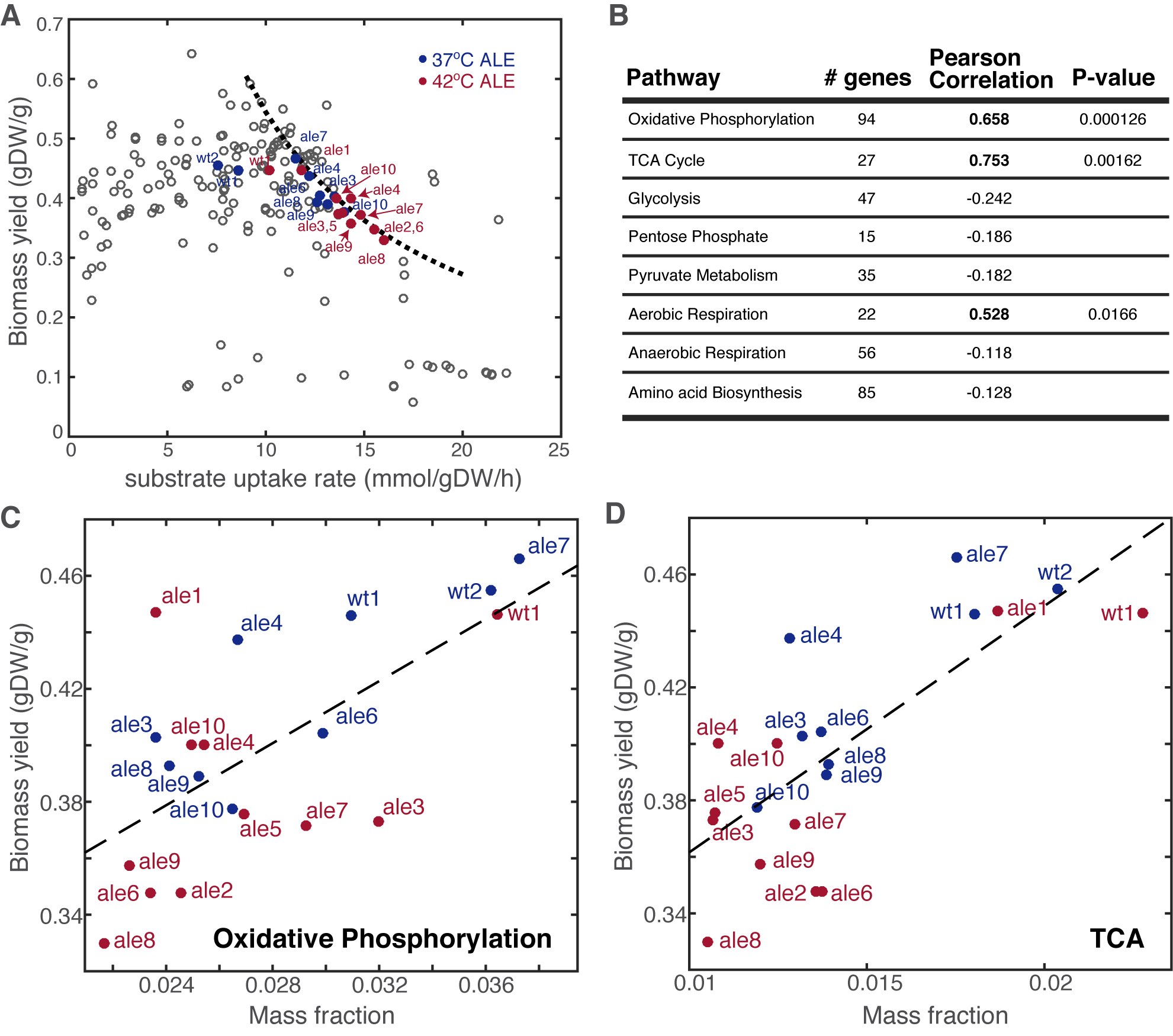

Supplement: S4 Fig — (A) Phenotypic data of 20 E. coli strains under glucose minimal medium are highlighted on the rate-yield space. Data includes two wild-type strains at 37°C (blue circle, labeled with “wt”), one wild-type strain at 42°C (red circle, labeled with “wt”), 7 strains evolved at 37°C (blue circles, labeled with “ale” and the strain number), and 10 strains evolved at 42°C (red circles, labeled with “ale” and the strain number). (B) Mass fraction of the representative pathway is calculated using all genes involved in the corresponding pathway. The Pearson correlation between the biomass yield and the mass fraction of each pathway is shown. Usage of three pathways that are related to aerobic respiration is significantly correlated with biomass yield (shown in bold, with their P-values listed). (C) Biomass yield plotted against the mass fraction of genes involved in oxidative phosphorylation. Each point corresponds to a strain, labeled as in (A). (D) Biomass yield plotted against the mass fraction of genes involved in TCA cycle. (TIF) [file pcbi.1008596.s006.tif]

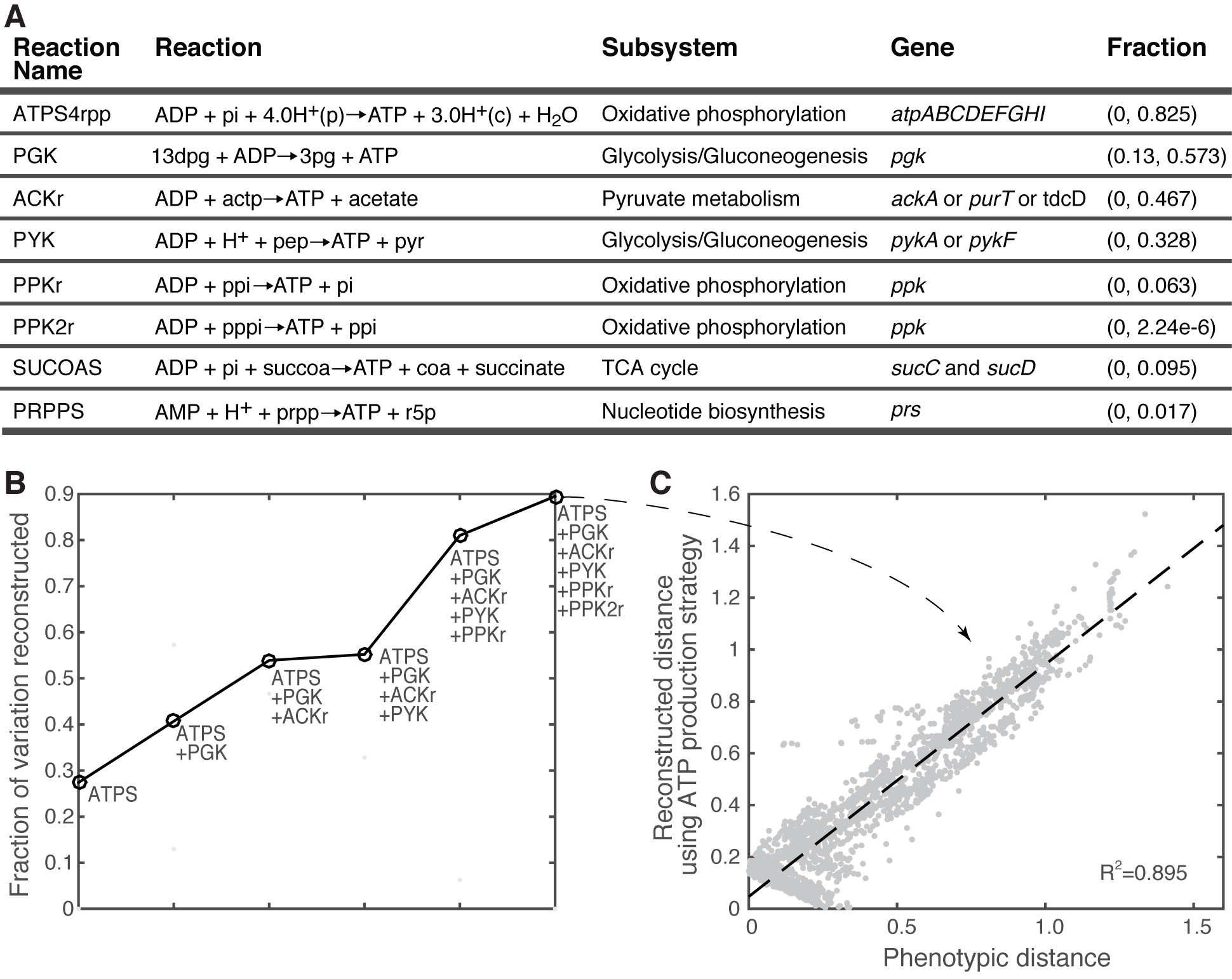

Supplement: S5 Fig — (A) Detailed information for the ATP-producing reactions in E. coli. Short and full name for the metabolites are listed as follows. 13dpg: 3-Phospho-D-glyceroyl phosphate; 3pg: 3-Phospho-D-glycerate; actp: acetyl phosphate; pyr: pyruvate; pep: phosphoenolpyruvate; succoa: succinyl-CoA; coa: coenzyme-A; prpp: 5-phospho-alpha-D-ribose 1-diphosphate; r5p: alpha-D-ribose 5-phosphate. The fraction of total ATP produced by each reaction varies significantly. The range of variation in the sampling simulations is indicated in the “Fraction” column. (B) Fraction of variations in the rate-yield phenotypic space explained by the indicated ATP-production reactions. 89.5% of the variations in phenotypic distance can be explained by the first six ATP-producing reactions. Among them, oxidative phosphorylation reactions ATPS4rpp and PPKr contributed the most. (C) Comparison between the actual phenotypic distance on the rate-yield plane and that reconstructed from the six ATP-producing reactions. A simulated phenotype is determined by a four-element vector containing the glucose uptake rate, acetate production rate, growth rate and biomass yield. Other typical phenotypic measurements are highly correlated with one or more chosen quantities, and are thus not included in the calculation. Then phenotypic distance is calculated as the Euclidean distance of this vector with respect to that of the wild-type solution at 37°C. Predictors of the stepwise linear regression are taken as the fraction of ATP produced by each reaction listed in (A). (TIF) [file pcbi.1008596.s007.tif]

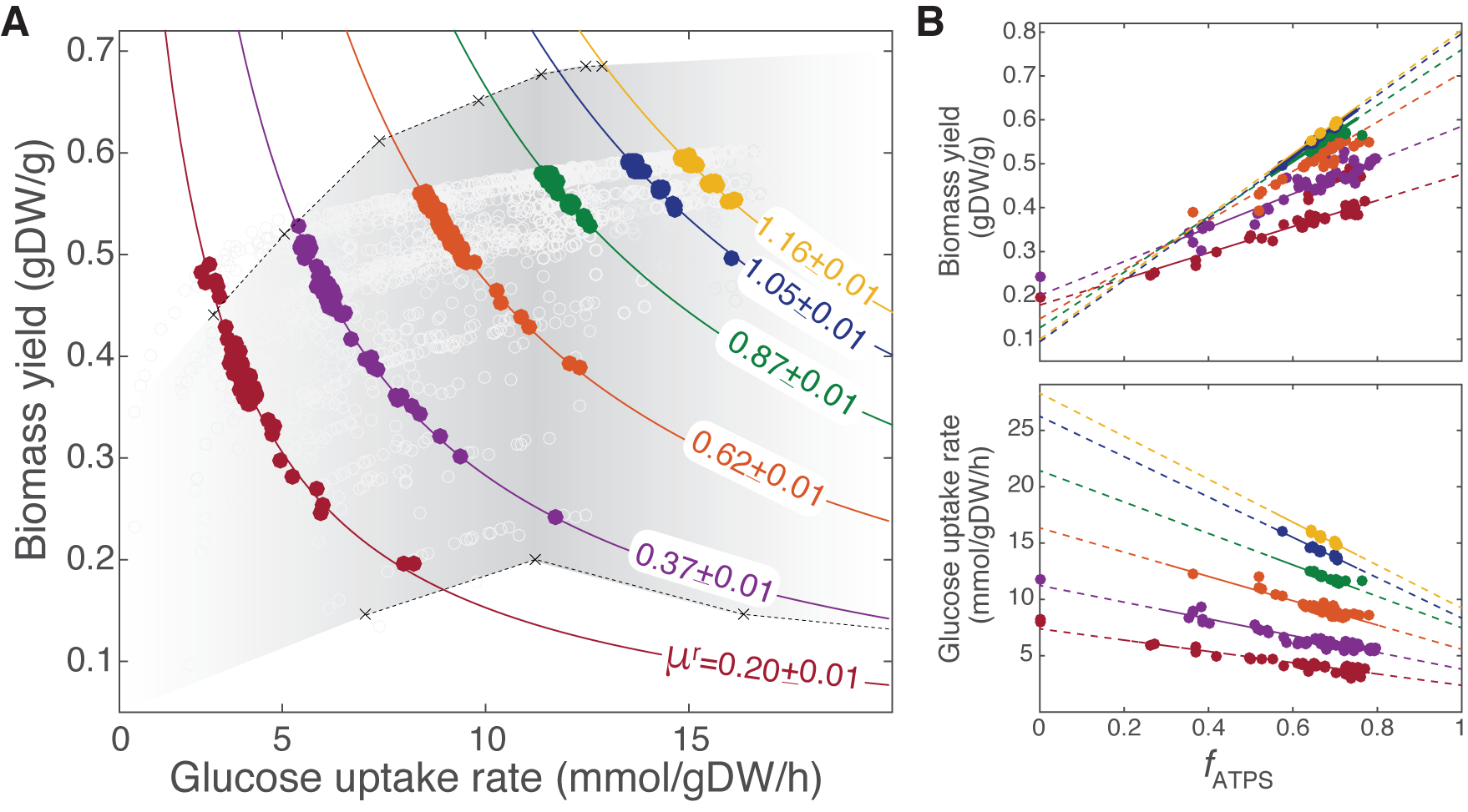

Supplement: S6 Fig — (A) Fitness distribution of the 2,200 simulated strains on the rate-yield plane (same as shown in Fig 1A). Simulated strains along six μ-isoclines, which are used in the subsequent analysis shown in panel B, are highlighted. Growth rates shown on the μ-isoclines are computed relative to the wild-type growth rate calculated at 37°C. (B) Along each μ-isocline, the calculated fraction of total cellular ATP produced by ATP synthase (fATPS) is linearly correlated with biomass yield (Y, top) and glucose uptake rate (qglc, bottom), with a positive and negative slope, respectively. The intercepts of these linear fitting at the minimum and maximum values of fATPS (0 and 0.83, respectively) provide a way to estimate the feasible range of qglc and Y. The estimations at each growth rate can be connected to draw the boundary of the rate-yield plane (gray shaded area in panel A). The accessible range of the phenotypic space generated this way encompasses the majority of data points from both experiments and model simulations. (TIF) [file pcbi.1008596.s008.tif]

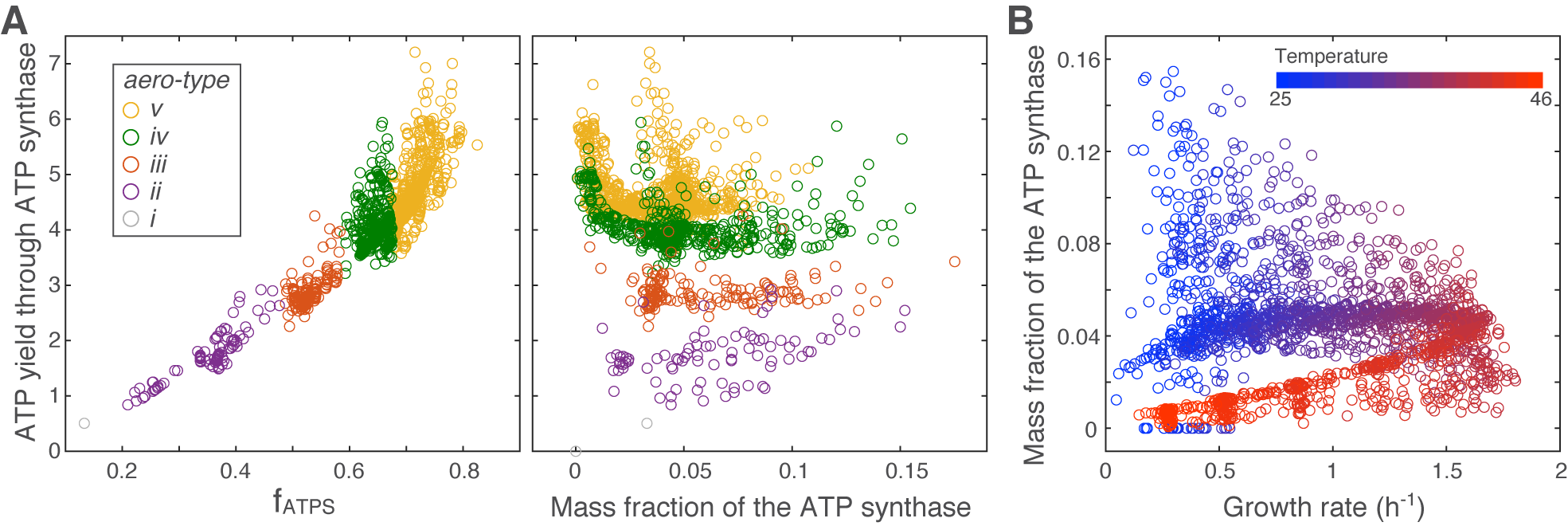

Supplement: S7 Fig — (A) ATP yield through the ATP synthase is positively correlated with fATPS (hence positively correlated with the aero-type as defined in the later Results sections), but not with the mass fraction of the ATP synthase in the proteome. (B) Expression of the ATP synthase is a function of the growth rate and temperature. (TIF) [file pcbi.1008596.s009.tif]

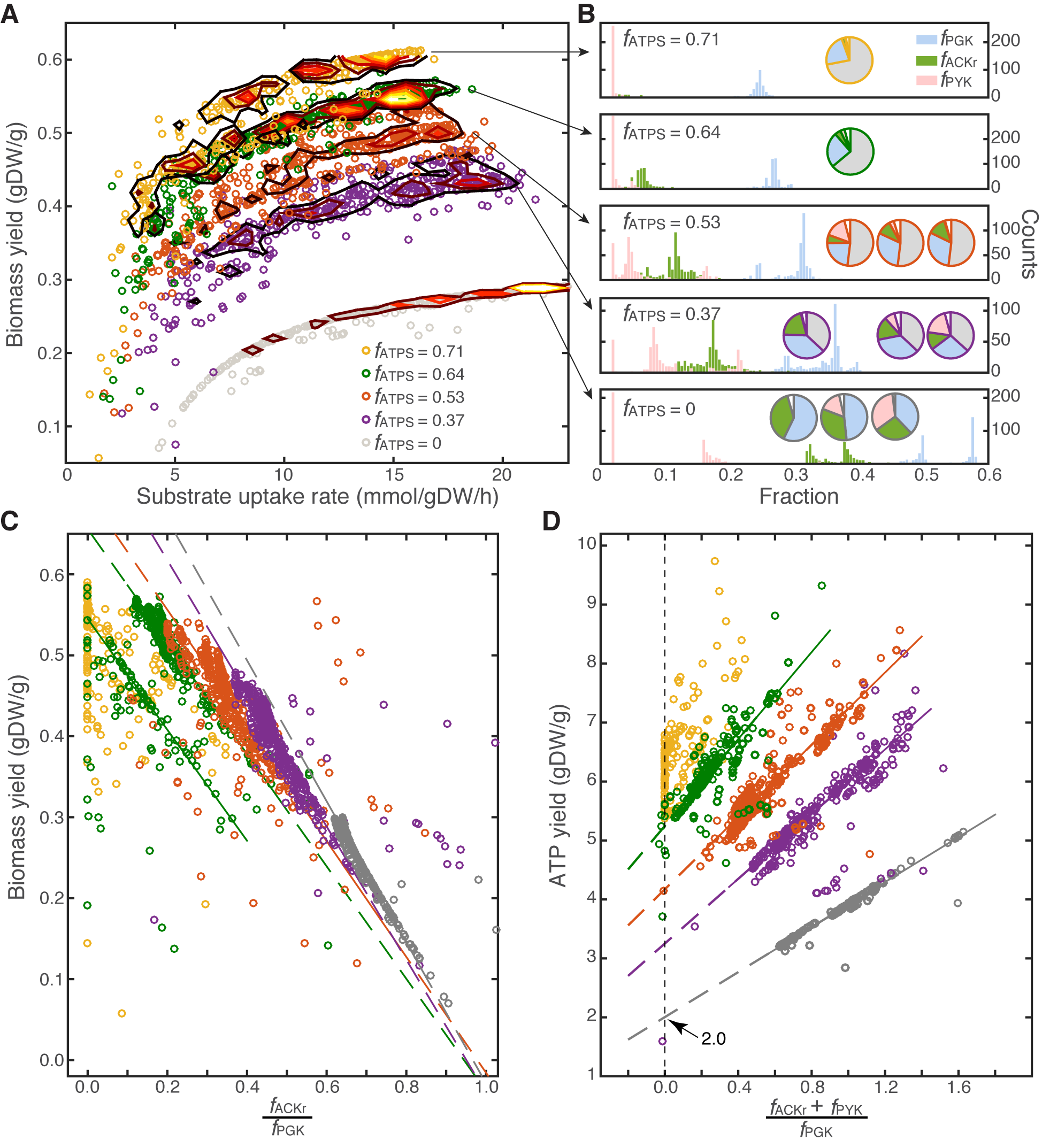

Supplement: S8 Fig — (A) Topography of the fitness landscape reconstructed from constrained sampling simulations at 5 most likely fATPS values: 0, 0.37, 0.53, 0.64 and 0.71. (B) Once fATPS is determined in the energy production strategy, finer structures can be seen on the phenotypic landscape. The distributions of fPGK, fACKr, and fPYK at each fixed fATPS value also show distinct multimodal distributions. The pie charts show the allowable energy production strategy that represents over 95% of the solutions at each fixed fATPS, all fractions shown have standard deviations smaller than 0.02. (C) At fixed fATPS, biomass yield is negatively correlated with the ratio fACKr/fPGK. Because acetate is secreted through the ACKr flux and no biomass is made, increase in fACKr reduces biomass yield. (D) At fixed fATPS, the overall ATP yield is positively correlated with the ratio (fACKr + fPYK)/fPGK. This ratio reflects the relative efficiency of all ATP-producing reactions in terms of ATP production per unit of substrate. The more inefficient reaction PGK is used (2 ATP per glucose uptake, which is reflected in the value of the fitting curve at (fACKr+fPYK)fPGK=0 and fATPS = 0), the lower the overall ATP yield is. As the yield of oxidative phosphorylation is much higher (∼34 ATP per glucose), the overall ATP yield increases with fATPS at fixed (fACKr + fPYK)/fPGK ratio. (TIF) [file pcbi.1008596.s010.tif]

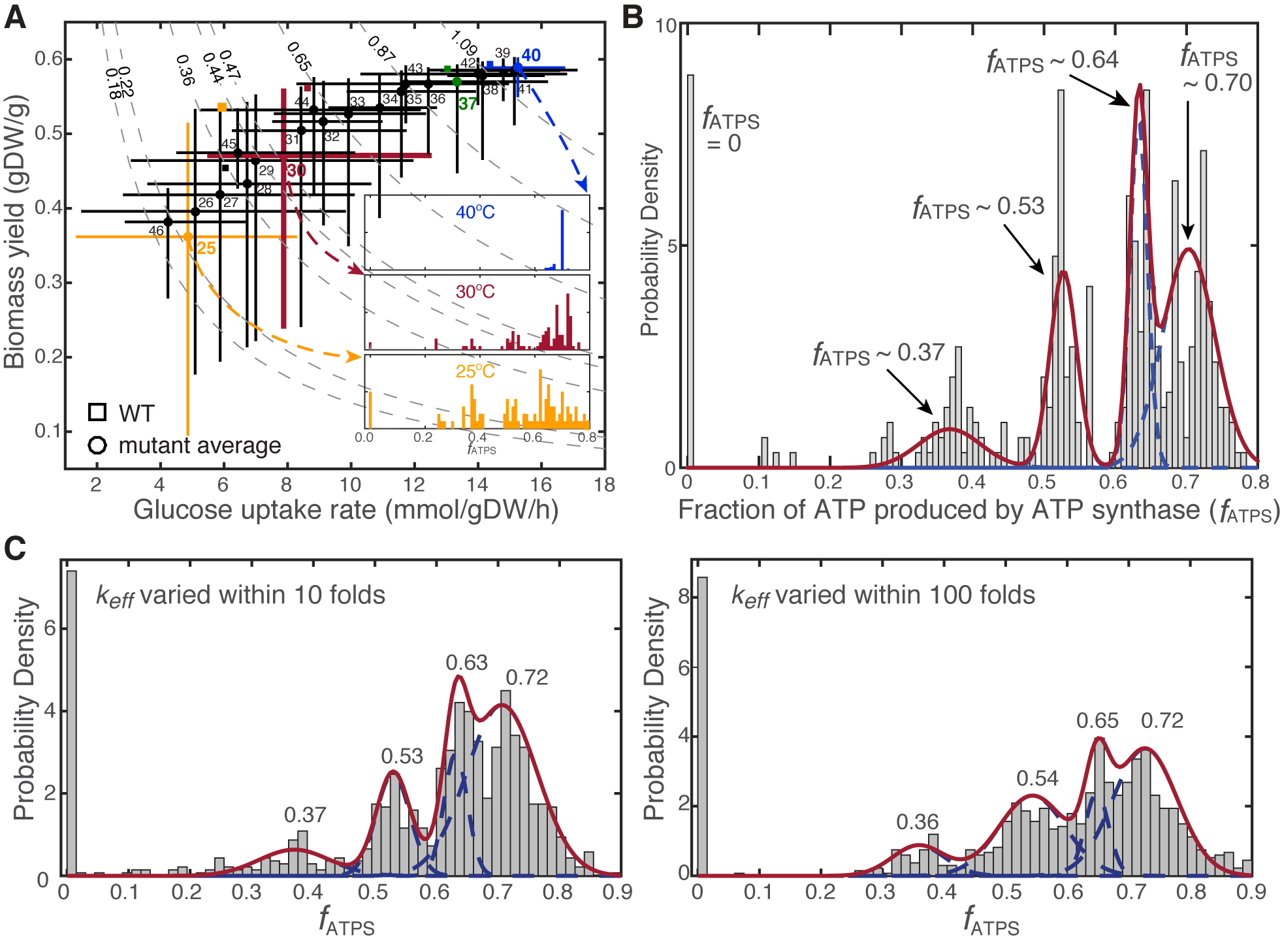

Supplement: S9 Fig — (A) The phenoptypic variations at each temperature. On this plot, the average values of Y and qglc calculated for the sampled strains at each temperature are denoted by a circle, then the range of accessible values indicated by horizontal and vertical lines going through the average. For T = 25°C, 30°C, 37°C and 40°C, the optimal wild-type phenotype (square) is shown for reference. For T = 25°C, 30°C, and 40°C, shift in the preferred aero-type is shown by the difference in fATPS distribution. Eight μ-isoclines are drawn, each labeled with the relative growth rate with respect to the simulated optimal WT growth rate at 37°C. Distribution of fATPS at 30°C best captures the features of the full distribution, thus we select this temperature for the down-stream analysis. (B) The fATPS distribution of the 368 sampling simulations performed at 30°C and selected growth rate. Fitting to a mixture of four Gaussian distributions shows consistency with the observed stratified distribution shown in Fig 1C. (C) fATPS value shows a similar multi-modal distribution as the maximum fold change in enzyme efficiency increases to 10 and 100 fold in the sampling simulation. (TIF) [file pcbi.1008596.s011.tif]

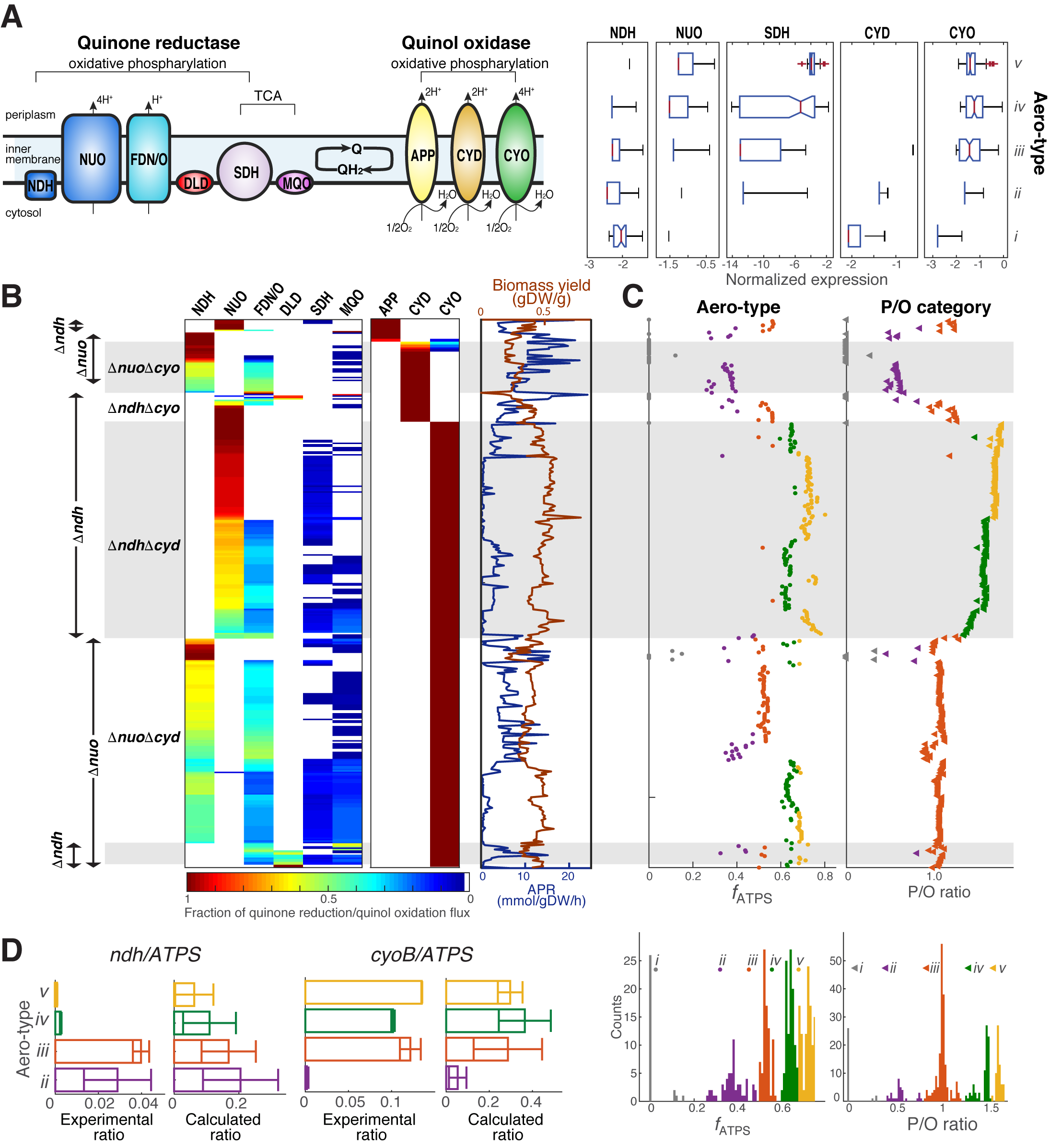

Supplement: S10 Fig — (A) Major protein complexes involved in quinone turnover in the sampling simulations. Formate dehydrogenase N and O catalyze the same reaction, hence are designated to the same complex (FDN/O) for simplicity. Box plot of the normalized expression for the indicated protein complexes shows differential usage of the ETC enzyme between different aero-types. To enable direct comparison, the calculated mass fraction of the enzyme complexes is normalized by the total mass fraction of all ribosomal proteins to remove bias coming from different growth rates. The central red line of the box plot shows the median, the bottom and top edges indicate the 25th and 75th percentiles, and whiskers extend to 1.5 times the interquartile range. Sample size in each aero-type is the same as in Fig 3D. (B) The activated ETC reactions in the 368 sampling simulations are shown with their relative contributions to the quinone reduction flux and quinol oxidation flux. The calculated biomass yield and acetate production rate are shown to the right, to represent the corresponding simulated phenotype. (C) fATPS and the P/O ratio are tentatively binned into five separate groups based on their multimodal distribution, and mapped to the optimal solutions shown in panel B. (D) Comparison of the experimental and simulated relative abundances of selected genes (ndh, cyoB) with respect to the ATP synthase. Length of the bar and error bar represent the average ratio and standard deviation for each aero-type as defined in Fig 4B for experiment, and in panel C for simulations. (TIF) [file pcbi.1008596.s012.tif]

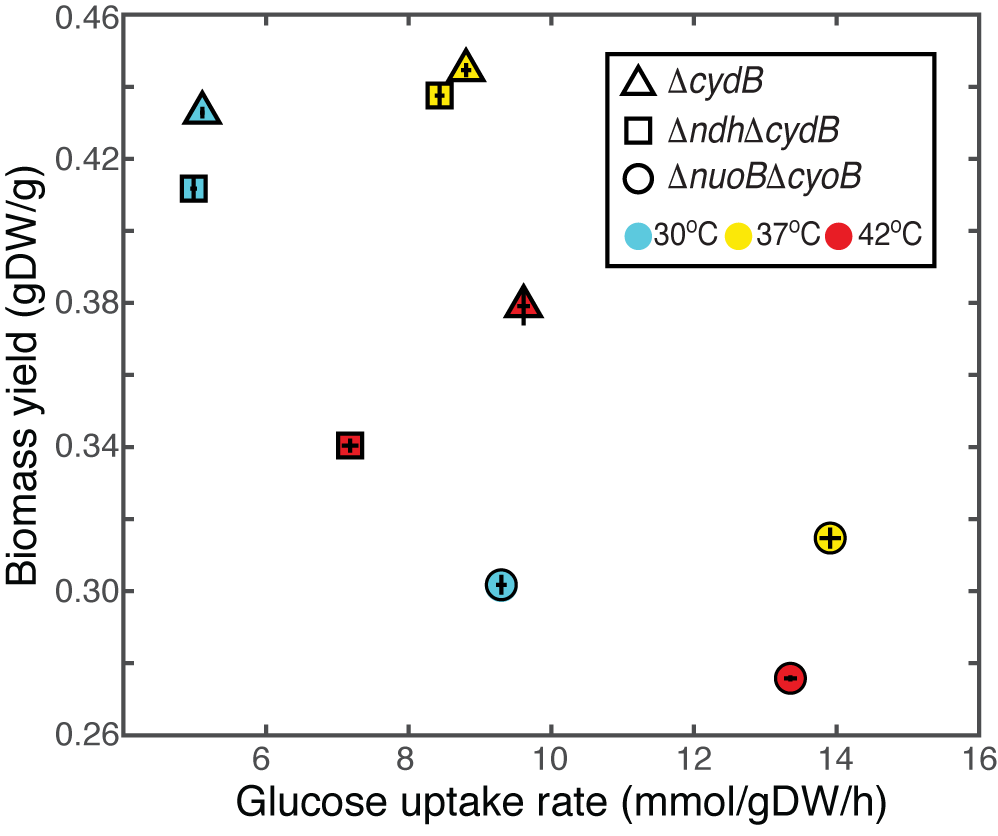

Supplement: S11 Fig — The ΔndhΔcydB, ΔcydB mutants were chosen to represent the higher aero-types v/iv, and the ΔnuoBΔcyoB mutant was chosen to represent a lower aero-type ii. Growth data at 30°C and 37°C nicely recapitulates the expected trend such that ΔndhΔcydB and ΔcydB stay in the region for aero-type iv and ΔnuoBΔcyoB in the region for aero-type ii. At 42°C, all three strains generate a lower biomass due to the temperature stress. However, they maintain well separated on the rate-yield plane representing the aero-type constraints caused by the removal of the respective ETC genes. Thus, the presented data supports the notion that the differential usage of the ETC genes determines the phenotypic aero-type of a cell. (TIF) [file pcbi.1008596.s013.tif]

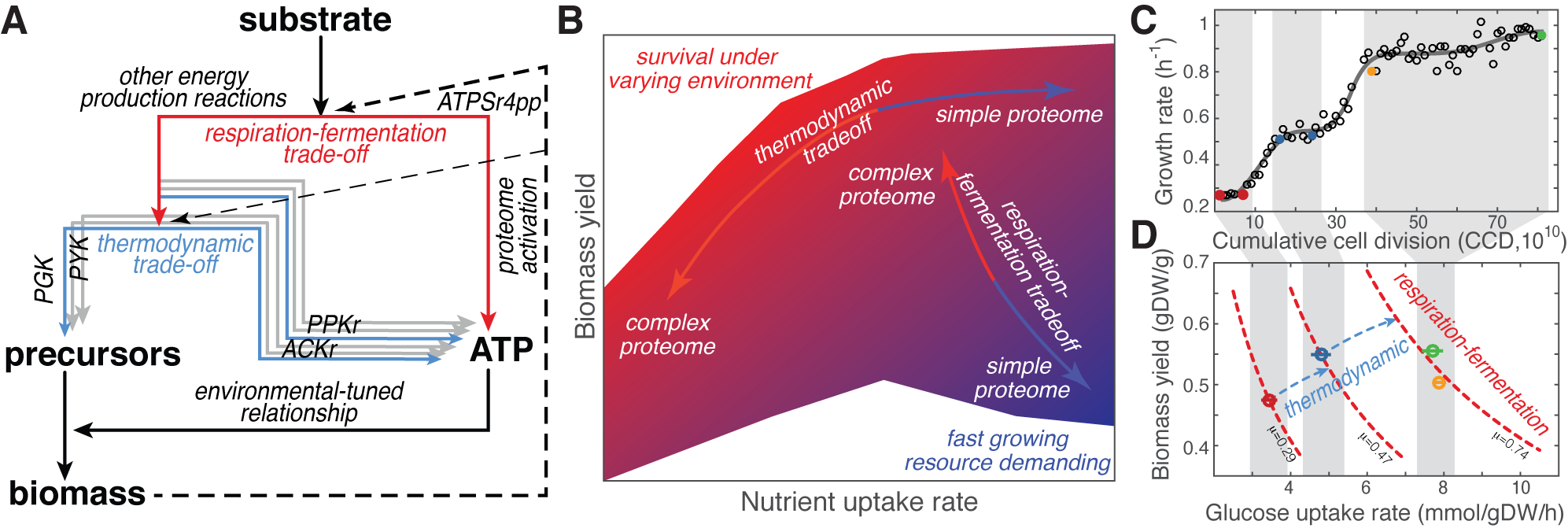

Supplement: S12 Fig — (A) The schematic of the proposed hierarchical energy production strategy. Blue and red arrows correspond to the thermodynamic and respiration-fermentation tradeoff, respectively. (B) A coarse-grained representation of the fitness landscape on the rate-yield plane. Color gradient indicates the level of proteome complexity, where blue represents the simpler proteome and red is the more complex proteome. (C) An example adaptive trajectory during the evolution of a pgi-deficient strain. (D) Intermediate evolutionary states were chosen at the indicated stages and characterized on the rate-yield plane. Four distinct genotypes were identified along the adaptive trajectory, indicated by red, blue, yellow, and green circles, respectively. Error bars indicate standard deviation of the biological duplicates. (TIF) [file pcbi.1008596.s014.tif]
