# Supplementary material for: Bacterial fitness landscapes stratify based on proteome allocation associated with discrete aero-types
Source: PLoS Comput Biol. 2021 Jan 19;17(1):e1008596. doi: 10.1371/journal.pcbi.1008596 (PMC7846111; doi:10.1371/journal.pcbi.1008596)
Supplement: S1 Table — (PDF) [file pcbi.1008596.s015.pdf]

S1 Table Protein Complexity for Selected Metabolic Pathways

Pathway	OxPhos	TCA	Glycolysis	Pyruvate	OxPPP
Total # reactions	51	14	24	8	12
# single-gene reaction	19	5	13	1	7
# multi-gene reaction	1	5	8	5	5
# single-complex reaction	15	6	3	0	0
# multi-complex reaction	16	0	0	2	0

Pathway names are shortened for compact display of the table. “OxPhos” stands for oxidative phosphorylation pathway, “Pyruvate” stands for pyruvate metabolism pathway, and “oxPPP” stands for oxidative branch of the pentose phosphate pathway.
