# Supplementary material for: Bacterial fitness landscapes stratify based on proteome allocation associated with discrete aero-types
Source: PLoS Comput Biol. 2021 Jan 19;17(1):e1008596. doi: 10.1371/journal.pcbi.1008596 (PMC7846111; doi:10.1371/journal.pcbi.1008596)
Supplement: S2 Table — (PDF) [file pcbi.1008596.s016.pdf]

S2 Table Phenotype Comparison of the ETC Knock-out Strains

Strain	μ	Y^a	q_{glc}	q_{ac}	q_{O_2}
Δndh	0.64±0.02	0.55	6.43±0.08	1.50±0.07	7.06±1.04
$\Delta cydB$	0.70^b	0.50	7.79±0.38	4.47±0.10	9.08±0.93
$\Delta nuoB$	0.69±0.01	0.44	8.69±0.09	6.50±0.14	12.27±0.42
$\Delta cyoB$	0.83^b	0.41	11.20±0.11	8.97±0.02	12.98±0.68
$\Delta ndh \Delta cydB$	0.61^b	0.46	7.35±0.24	4.14±0.10	8.67±1.07
$\Delta ndh \Delta cyoB$	0.62^b	0.43	7.97±0.12	7.50±0.18	10.26±0.63
$\Delta nuoB \Delta cydB$	0.62±0.01	0.45	7.65±0.21	5.17^b	10.29±0.53
$\Delta nuoB \Delta cyoB$	0.70±0.02	0.35	11.09±0.99	11.27±0.89	15.84±0.81
ECOM^c	0.40±0.013	0.11±0.006	20.31±1.50	3.55±0.50	-

^{a} Biomass yield (Y) is calculated using the averaged growth rate (μ) and glucose uptake rate (q_{glc}), with no uncertainty from biological replicates.
^{b} Difference between biological replicates is smaller than 0.01.
^{c} Values in this row are averaged from ten evolved strains described in Portnoy et al. Uncertainty is reported by standard deviation.
