# Supplementary material for: Bacterial fitness landscapes stratify based on proteome allocation associated with discrete aero-types
Source: PLoS Comput Biol. 2021 Jan 19;17(1):e1008596. doi: 10.1371/journal.pcbi.1008596 (PMC7846111; doi:10.1371/journal.pcbi.1008596)
Supplement: S3 Table — (PDF) [file pcbi.1008596.s017.pdf]

S3 Table: Sequence of the confirmation primers

Kanamycin specific primers	
k1[a]	CAGTCATAGCCGAATAGCCT
k2[a]	CGGTGCCCTGAATGAACTGC
Locus specific primers	
ndh(U)[b]	TGTTTTTTGATCTCACCCGG
ndh(D)[b]	TCCTTCTTTGCAGTTATGCC
cydB(U)	CGAACTCGTCACTGACCGCA
cydB(D)	CCATCACGAAGGAAAGCGCC
nuoB(U)	CATGGTCAACCTCTATCCGC
nuoB(D)	TCCGGCCCAAAACGGTTGCG
cyoB(U)	CTAGCGAATACAACCAGGTG
cyoB(D)	CCAGAACGAACGGCAGTTCG

[a] For detailed explanation of the primers k1 and k2, see Baba et al.
[b] U: upstream primer; D: downstream primer.
